# Alternative application of an affinity purification tag: hexahistidines in ester hydrolysis

**DOI:** 10.1038/s41598-017-15310-y

**Published:** 2017-11-07

**Authors:** Lise Schoonen, Kayleigh S. van Esterik, Chunqiu Zhang, Rein V. Ulijn, Roeland J. M. Nolte, Jan C. M. van Hest

**Affiliations:** 1 Department of Bio-Organic Chemistry, Eindhoven University of Technology, PO Box 513 (STO 3.31), 5600 MB Eindhoven Eindhoven, The Netherlands; 2 Department of Bio-Organic Chemistry, Institute for Molecules and Materials, Radboud University, Heyendaalseweg 135, 6525 AJ Nijmegen, The Netherlands; 3 Advanced Science Research Center, City University of New York, 85 St Nicholas Terrace, New York, 10031 USA

## Abstract

Hexahistidines are very common tags used in the affinity chromatography purification of recombinant proteins. Although these tags are solely applied for their metal-binding properties, we found that they are also able to perform ester hydrolysis when attached to a protein. For instance, green fluorescent protein (GFP) and the cowpea chlorotic mottle virus (CCMV) are able to perform catalysis after introduction of the His-tag. By attaching a His-tag to an enzyme, a dual-functional catalyst was created, that can perform a two-step cascade reaction. These findings show that the catalytic properties of the hexahistidine tag should be taken into consideration when choosing a suitable protein purification tag.

## Introduction

Hexahistidine tags (His-tags) are among the most popular affinity purification tags for recombinant proteins^1,2^. The advantages of these tags over other purification tags for recombinant protein purification are that: (i) the tag can be incorporated at both the C- and the N-terminus of a protein, (ii) the tags have little interference with the protein structure due to their small size and neutral charge at physiological pH values, and (iii) the proteins can be eluted under mild conditions after affinity chromatography, allowing them to retain their biological activity.

In His-tags, the metal-coordinating properties of histidines are exploited. Metals such as nickel, cobalt and copper are known to efficiently bind histidines. This is demonstrated in enzymes, for instance, where histidines can be used for metal ion binding in the active sites. Histidines, being among the most versatile naturally occurring amino acids, are also regularly found in the catalytic sites of enzymes, whether or not as a part of a catalytic triad^3,4^. Here, they can play a role in general acid-base catalysis. The versatility of this amino acid stems from the imidazole group in its side chain. This group is amphoteric and able to lose a proton via its pros nitrogen atom, whereas it can accept a proton via the nitrogen in the tele position at pH values close to pH 7^5^. Its unique properties make that imidazoles, and thus histidines, can take part in both nucleophilic and base catalysis, in addition to metal coordination.

It is already known for a long time that imidazole is able to catalyze the hydrolysis of *p*-nitrophenyl acetate (*p*-NPA)^6^. Since the first report in 1956, the imidazole group in histidine has been applied as a catalytic function, e.g. when present in polymers, peptide bundles or micelles^7–12^. Furthermore, it was shown that imidazoles, and hence histidines, can catalyze other types of reactions, such as aldol reactions and RNA cleavage^13–17^.

We reasoned that the introduction of a His-tag in a protein for purification purposes might not be as innocent as previously thought. Since histidines have such unique properties, as a consequence of their side chain imidazole functionalities, the introduction of six copies of this amino acid could have serious implications on the properties of the resulting tagged protein. Here, we will show that the effects of the introduction of a His-tag are indeed not negligible, in terms of the catalytic properties of the resulting protein construct. Our findings show that the catalytic properties of His-tags should be taken into consideration, in addition to their advantageous properties, when choosing a protein purification tag for a specific application.

## Results and Discussion

### Esterase activity of His-tags

**Figure 1:**
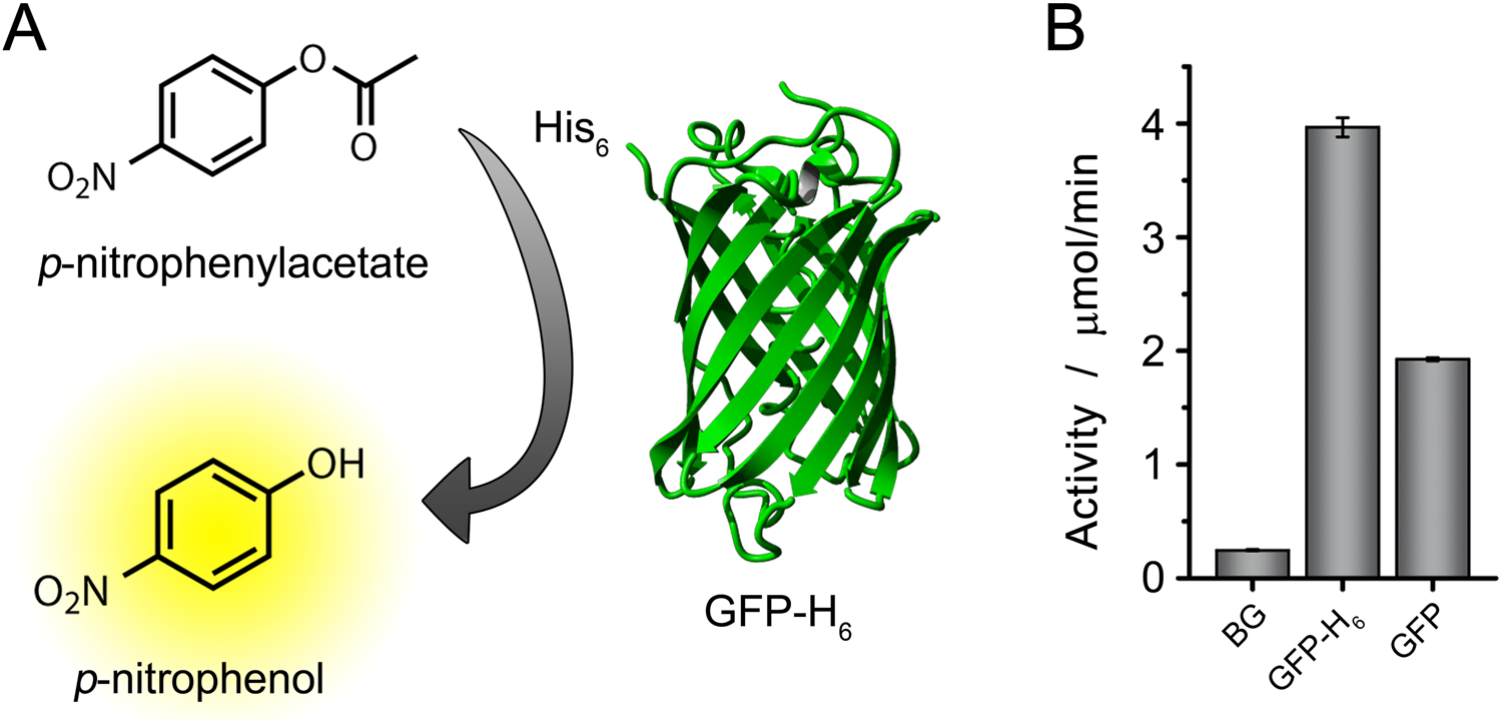
(**A**) Schematic representation of the His_6_-GFP catalyzed conversion of p-nitrophenyl acetate (p-NPA) to p-nitrophenol (p-NP). (**B**) Activity of His_6_-GFP in the conversion of p-NPA to p-NP, compared to background hydrolysis (BG) and the activity of GFP after cleavage of the His-tag.

Green fluorescent protein (GFP) was chosen as a model enzyme to study the catalytic properties of the His-tag. His_6_-GFP was dissolved in PBS buffer and the activity of the His-tag was measured by recording the conversion of *p*-NPA to *p*-nitrophenol (*p*-NP) and acetic acid, using a spectrophotometric assay (Fig. 1A). It was found that His_6_-GFP is indeed able to hydrolyze *p*-NPA to *p*-NP, and that its activity can easily be distinguished from the background hydrolysis of *p*-NPA (Fig. 1B). It was also observed that the activity is linearly dependent on the His_6_-GFP concentration, supporting the fact that His_6_-GFP indeed acts as the catalyst in this reaction (Figure S1). The activity of His_6_-GFP was compared to the activity of GFP, after cleavage of the His-tag using TEV protease. It was found that more than half of the activity was lost upon cleavage of the His-tag (Fig. 1B). We believe the activity of GFP stems from the nine residual histidines in its endogenous sequence, which might also act as catalysts. Comparing the activity of His_6_-GFP to the activity of esterase *Candida antarctica* lipase B (CalB) in PBS buffer, it was found that the activity of His_6_-GFP is a factor of approximately 3000 lower (~14 µmol/min/mg for His_6_-GFP vs. ~40.000 µmol/min/mg for CalB) (Figure S2).

**Figure 2:**
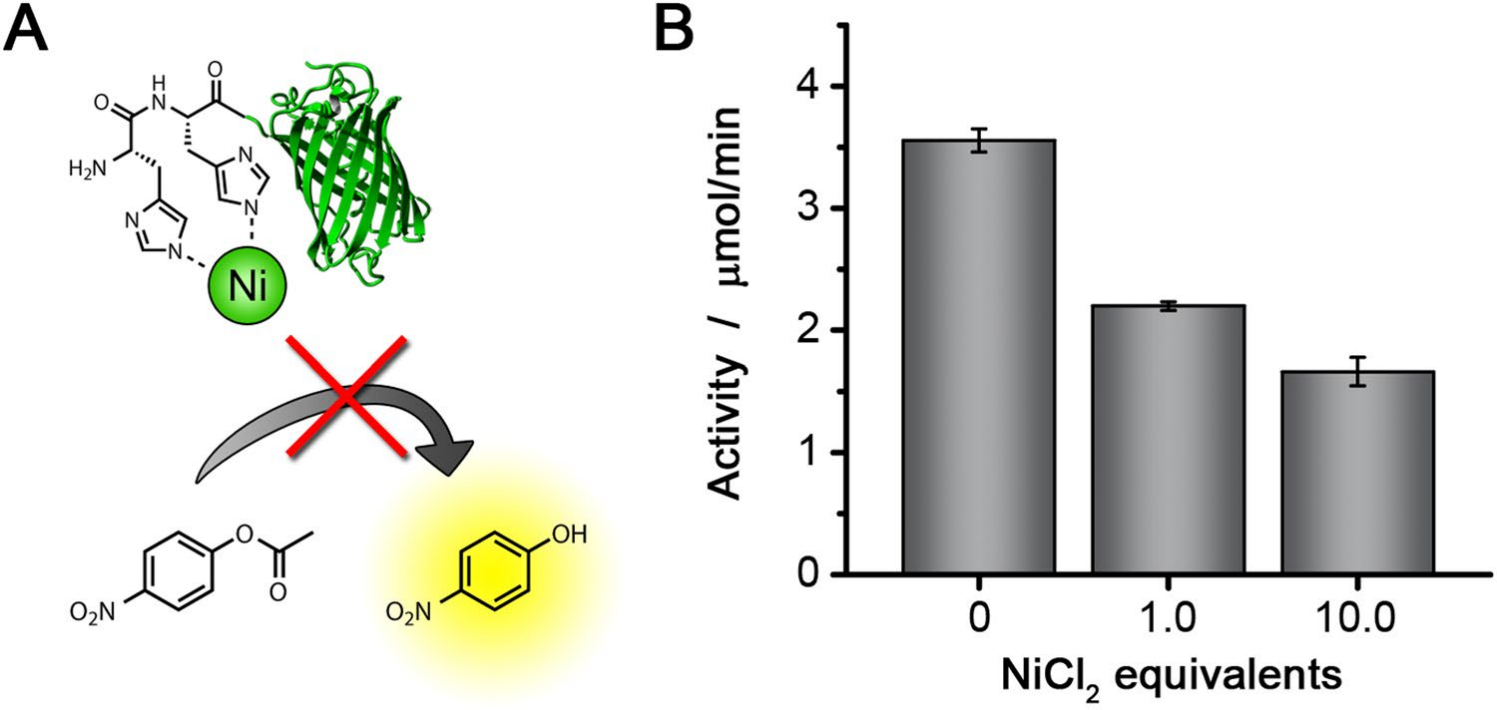
(**A**) Schematic representation of inhibition of the esterase activity of His_6_-GFP by coordination of nickel ions to the His-tag. (**B**) Activity of His_6_-GFP after incubation with different equivalents of NiCl_2_. The conversion of *p*-NPA to *p*-NP was measured.

To provide definite proof that the His-tags are responsible for the observed activity of His_6_-GFP, we performed the same activity assay, but now the protein was first incubated with varying equivalents of NiCl_2_. If the imidazole groups of the histidines are responsible for the conversion, binding of nickel would block the catalytically active groups, inhibiting the reaction (Fig. 2). It was observed that the addition of 10 equivalents of NiCl_2_ reduced the activity of His_6_-GFP significantly.

### Scope of His-tag catalysis

**Figure 3:**
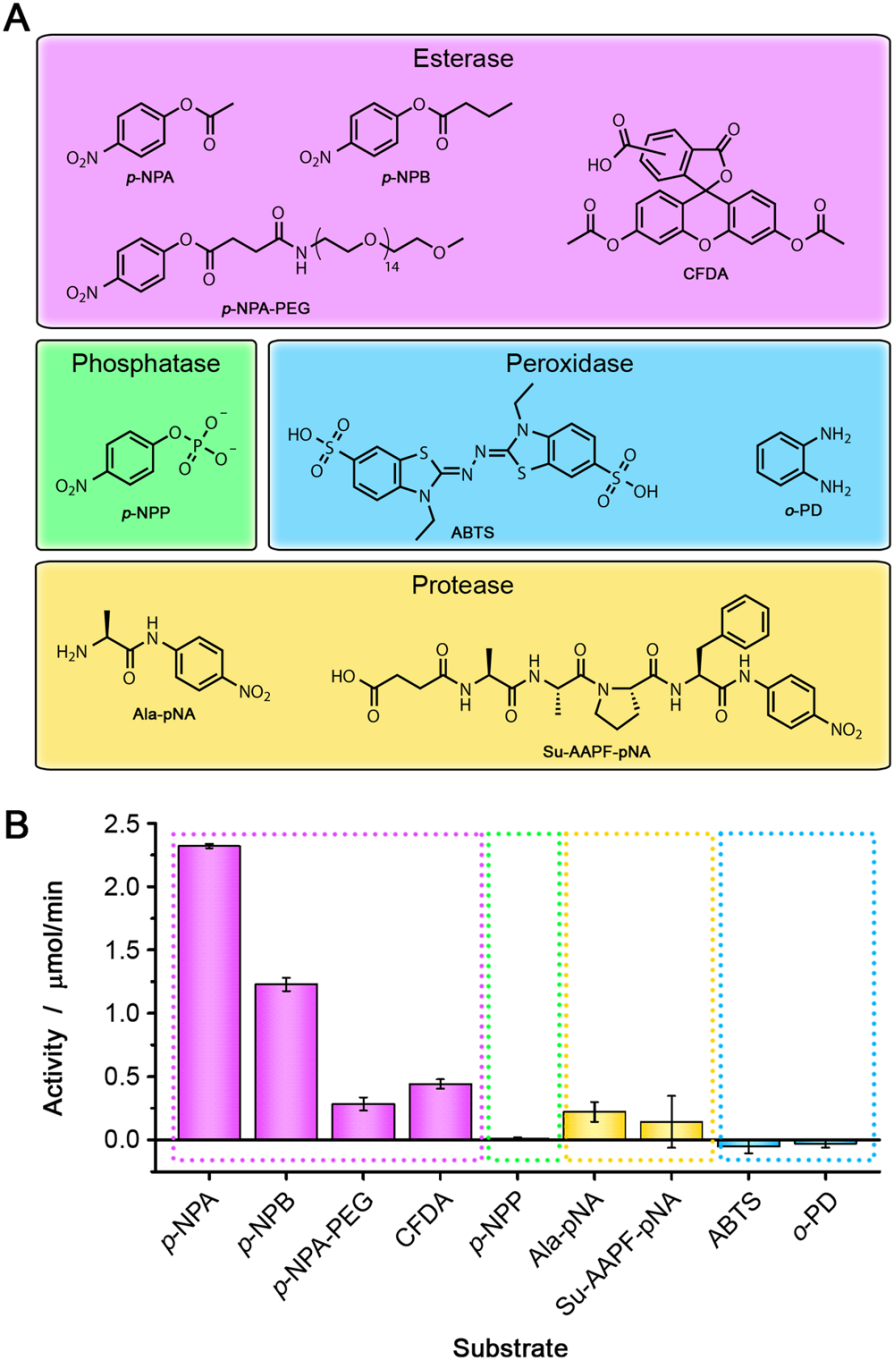
(**A**) Different classes of substrates that were tested in the His_6_-GFP activity assay. (**B**) Activity of His_6_-GFP upon incubation with different substrates. H_2_O_2_ was added to the reactions with ABTS and *o*-PD. The slope of the background reaction was subtracted from the slope of the catalytic reaction. In all cases, the background reaction was less than 10% of the activity of the catalyzed reaction.

Next, we investigated whether the His-tag can also hydrolyze other esterase substrates. To this end, we tested *p*-nitrophenyl butyrate (*p*-NPB), polyethylene glycol MW 800-modified *p*-NPA (*p*-NPA-PEG) and carboxyfluorescein diacetate (CFDA) (Fig. 3). In all cases, hydrolysis to the corresponding alcohols was observed, and a slower reaction was observed for the larger substrates. In a previous paper, we observed a similar effect for CalB in experiments that were performed under very similar reaction conditions: large substrates were converted more slowly than small substrates^18^.

After having established that the His-tag is an efficient catalyst in ester hydrolysis, we studied the scope of its catalytic activity. Structurally similar reactions were investigated, e.g. the hydrolysis of phosphate monoesters (phosphatase activity), reactions that are often catalyzed by enzymes with a histidine-containing catalytic triad in their active sites, e.g. proteolysis (protease activity), and reactions catalyzed by enzymes in which histidine is involved in metal-binding, e.g. hydrogen peroxide oxidation (peroxidase activity) (Fig. 3). Surprisingly, the activity of His_6_-GFP in the anilide hydrolysis reactions was comparable to the activity in some of the esterase reactions, which is of interest for future studies.

### Investigation of cooperativity in His-tag catalysis

**Figure 4:**
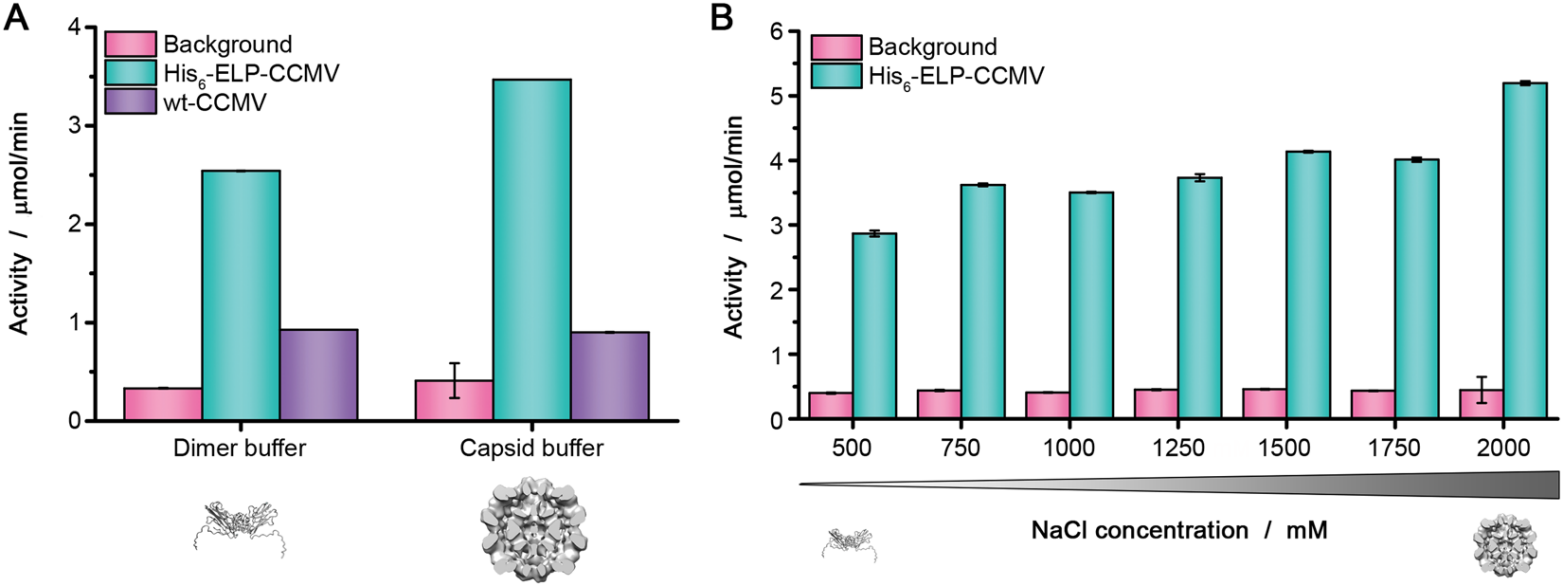
(**A**) Activity of His_6_-ELP-CCMV and wt-CCMV in the conversion of *p*-NPA to *p*-NP, both in dimer buffer and capsid buffer. (**B**) Activity of His_6_-ELP-CCMV in dimer buffer with increasing NaCl concentrations.

To investigate whether the activity of the His-tags is enhanced by the close proximity of several imidazole groups in the adjacent histidine residues, we decided to study the activity of a His-tagged self-assembling protein. We chose the cowpea chlorotic mottle virus (CCMV) capsid protein for this purpose. In particular, we used a block-copolymer variant of the capsid protein, which is equipped with an elastin-like polypeptide (ELP) at its N-terminus. The introduction of this stimulus responsive polymer resulted in new assembly properties of the His_6_-ELP-CCMV capsid protein, as we showed before^19^. With this modified protein, capsid formation can be induced by increasing the salt concentration or varying the temperature, aside from the pH-dependent assembly behavior. We used His_6_-ELP-CCMV as a catalyst in the conversion of *p*-NPA to *p*-NP, both in the dimer state and after salt-induced assembly of the capsids (Fig. 4A). As a control, the non-His-tagged wild-type CCMV capsid protein (wt-CCMV) was used. Significant activity was observed for His_6_-ELP-CCMV compared to the background hydrolysis. Similar to His_6_-GFP, its activity depends on the His_6_-ELP-CCMV concentration and can be inhibited by the addition of NiCl_2_ (Figures S3 and S4). The activity in the capsid buffer, containing 2000 mM NaCl, was higher than in the dimer buffer with 500 mM NaCl. In order to explain this trend, we investigated the effect of salt concentration on the catalysis of His_6_-ELP-CCMV (Fig. 4B). We found that the activity is indeed salt concentration dependent. An increase in activity was especially observed between 1750 mM and 2000 mM NaCl. Based on DLS analysis of the assembly state of His_6_-ELP-CCMV at different salt concentrations, we can conclude that this coincides with the formation of capsids around 1.7 M NaCl (Figure S5). In separate experiments, it was found that the activity of His_6_-GFP does not depend similarly on the NaCl concentration (Figure S6).

The control protein without a His-tag, wt-CCMV, shows some activity in the two buffers that were used (dimer buffer and capsid buffer), compared to the background hydrolysis of *p*-NPA (Fig. 4A). This small activity is most probably due to the two endogenous histidine residues of wt-CCMV. It should be noted that wt-CCMV does not form capsids when the salt concentration is increased, i.e. when going from dimer buffer to capsid buffer. This might be the reason why we do not observe an increase in activity at higher NaCl concentrations for wt-CCMV.

### Activity assays with free histidine library

**Figure 5:**
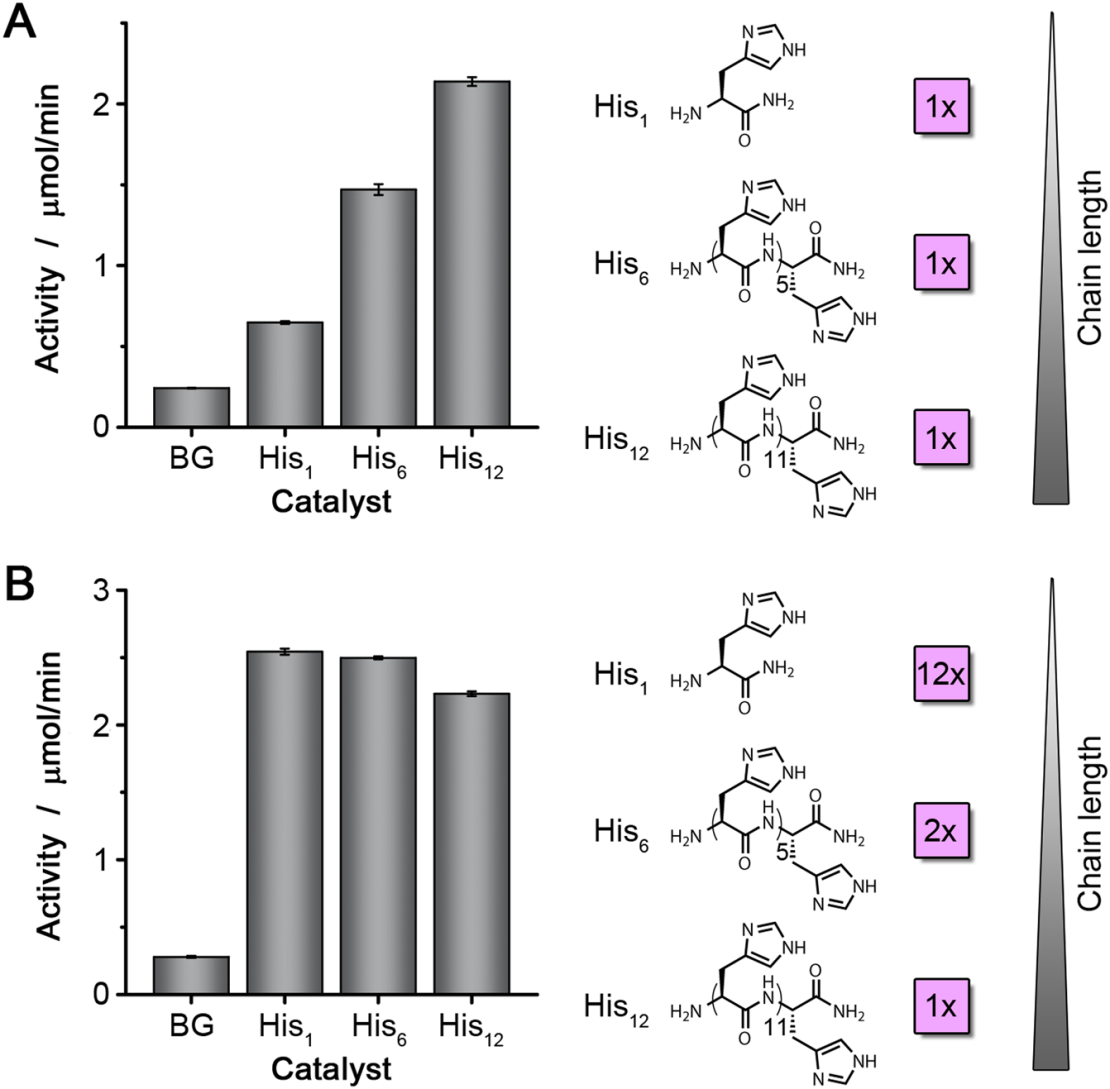
Activity assays with histidine peptide library. (**A**) Comparison of the activity of equimolar amounts of His_1_, His_6_, and His_12_ peptides. (**B**) Comparison of the activity of His_1_, His_6_, and His_12_ peptides, when the same histidine concentration was present in each reaction.

To investigate the histidine catalysis further, we examined the esterase activity of a small library of histidine derivatives, consisting of H-His-NH_2_, H-His_6_-NH_2_ and H-His_12_-NH_2_ (Fig. 5). It was found that longer histidine peptides show higher activity, when equimolar amounts of the peptides were used. When the concentrations of the catalyst were adjusted, such that in each solution the same amounts of imidazole groups were present, the differences in catalytic activity were very small. This shows that the catalysis is not cooperative: more histidines in close proximity to each other do not necessarily increase the activity.

His_6_ was found to be approximately 2–3 times less active than His_6_-GFP and His_6_-ELP-CCMV, when used at the same concentrations (Figure S7). Thus, in the case of these proteins, the protein environment might have a positive effect on the catalytic activity of a His-tag. A possible explanation for this effect could be the nonspecific binding of the substrate to the protein, resulting in a higher local substrate concentration. This observation might also explain the increase that is observed when going from CCMV dimers to capsids: the His-tags are much more surrounded by protein material and the hydrophobic aggregated ELPs in the capsid state than in the dimer state. Another explanation could be the influence of other types of neighboring residues, as it was shown previously that the activity of histidine-containing oligopeptides is sequence dependent^20^.

### Application of His-tag catalysis in a dual-active enzyme

**Figure 6:**
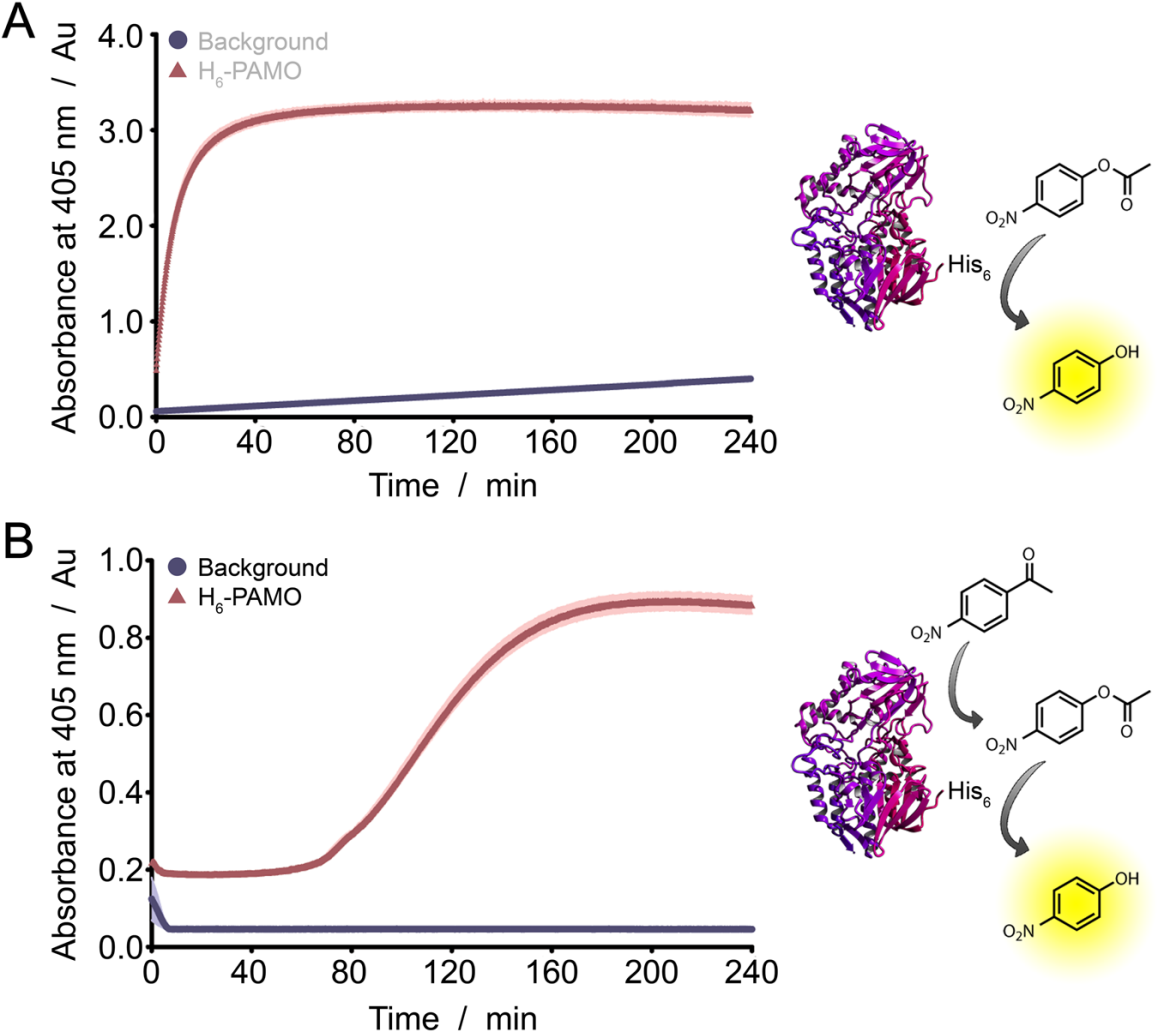
(**A**) Activity of His_6_-PAMO in the conversion of *p*-NPA to *p*-NP. (**B**) Activity of His_6_-PAMO in the cascade conversion of *p*-nitroacetophenone to *p*-NP via *p*-NPA.

Finally, we investigated whether we could convert an enzyme with one active site into a dual-active enzyme by introducing a His-tag. To this end, we expressed His-tagged phenylacetone monooxygenase (His_6_-PAMO), which is an oxidoreductase that normally catalyzes the conversion of phenylacetone to benzyl acetate^21^. We envisioned to use His_6_-PAMO in a cascade reaction, i.e. the conversion of *p*-nitroacetophenone to *p*-NP, via *p*-NPA. First, we tested whether the His-tag of His_6_-PAMO could catalyze the second step of this cascade (Fig. 6A). It was found that this reaction proceeds well; full conversion was reached after a reaction time of approximately 40 minutes. Some background reaction was observed, due to the spontaneous hydrolysis of *p*-NPA to *p*-NP in aqueous environments. Next, His_6_-PAMO was investigated as a dual-active enzyme in a cascade reaction, by introducing *p*-nitroacetophenone as the substrate. As expected, we observed the formation of *p*-NP with a delay, indicating the formation of an intermediate product. In this case, no background reaction was observed, as expected, because this starting material is not prone to spontaneous hydrolysis in aqueous environments. Notably, the final absorbance in this case was somewhat lower than for the conversion of *p*-NPA to *p*-NP. This might be a consequence of imine formation between the starting material *p*-nitroacetophenone and amine functions in the protein, resulting in a lower effective substrate concentration and hence a lower final product concentration. As a control, His_6_-PAMO was incubated with 10 equivalents of NiCl_2_ before the addition of *p*-nitroacetophenone (Figure S8). As expected, slower *p*-NP formation was observed due to inhibition of the His-tag activity due to nickel binding.

## Conclusions

His-tags are very often attached to recombinant proteins for purification purposes. In the abovementioned examples, we have shown that this is not without consequence. In fact, these proteins may now acquire catalytic properties, due to the clustering of several imidazole groups at the N- or C-terminus of the protein. More specifically, different types of esters can be hydrolyzed by the His-tags. Additionally, preliminary studies have indicated that His-tags might also act as proteases in peptide cleavage, which may be worth investigating further.

By studying the activity of a His-tagged self-assembling protein and a small library of histidine peptides, it was found that the surrounding protein or neighboring residues positively influence the catalysis performed by the His-tag. Finally, we showed that by providing an enzyme with a His-tag, its catalytic properties can be modified such that it can now catalyze a two-step cascade reaction.

We believe that the implications of the findings described in this paper are two-fold: (i) it might explain the unexpected background reactions that are sometimes observed when performing hydrolysis reactions in the presence of His-tagged proteins and (ii) this phenomenon can be used to our advantage, e.g. ester hydrolysis activity can be added to the catalytic properties of an enzyme, leading to a two- or even multi-step (enzymatic) reaction catalyzed by a single biocatalyst.

## Methods

### Materials

Ampicillin was purchased from MP Biomedicals. Chloramphenicol, *p*-nitrophenol, *p*-nitrophenyl acetate, *p*-nitrophenyl butyrate, Ala-pNA and Su-AAPF-pNa were obtained from Sigma-Aldrich. CFDA was purchased from VWR International. ABTS was obtained from Fluka. H-His-NH_2_ was purchased from Bio Connect. H-His_6_-NH_2_ and H-His_12_-NH_2_ were ordered at Pepscan. *p*-NPA-PEG was synthesized as described previously^18^. Isopropyl β-D-1-thiogalactopyranoside (IPTG) was purchased from Acros. Ni-NTA agarose beads were obtained from Qiagen. NiCl_2_ was purchased from Alfa Chemicals. *p*-Nitroacetophenone was obtained from Merck.

### Buffers

PBS buffer: 10 mM Na_2_HPO_4_, 1.8 mM KH_2_PO_4_, 137 mM NaCl, 2.7 mM KCl, pH 7.2–7.4 pH-induced assembly buffer: 50 mM NaOAc, 500 mM NaCl, 10 mM MgCl_2_, 1 mM EDTA, pH 5.0 Salt-induced assembly buffer/capsid buffer: 50 mM Tris·HCl, 2 M NaCl, 10 mM MgCl_2_, 1 mM EDTA, pH 7.5 Dimer buffer: 50 mM Tris·HCl, 500 mM NaCl, 10 mM MgCl_2_, 1 mM EDTA, pH 7.5 RNA buffer: 50 mM Tris·HCl, 500 mM CaCl_2_, 1 mM DTT, pH 7.5 Clean buffer: 50 mM Tris·HCl, 500 mM NaCl, 1 mM DTT, pH 7.5 Storage buffer: 50 mM NaOAc, 1000 mM NaCl, 1 mM NaN_3_, pH 5.0 PAMO buffer: 50 mM Tris·HCl, pH 7.5 CalB buffer: 50 mM NaH_2_PO_4_, 150 mM NaCl, pH 7.0

### UV-vis absorbance measurements

Protein concentrations were measured on a Varian Cary 50 Conc UV-vis spectrometer using a quartz cuvette with a path length of 3 mm. Protein concentrations were calculated using the theoretical extinction coefficients^22^. Samples were centrifuged prior to the measurements.

### Mass spectrometry

Protein mass characterization was performed by electrospray ionization time-of-flight (ESI-TOF) on a JEOL AccuTOF CS. Deconvoluted mass spectra were obtained using MagTran 1.03 b2. Isotopically averaged molecular weights were calculated using the ‘Protein Calculator v3.4′ at http://protcalc.sourceforge.net. Protein samples were desalted by spin filtration with MQ (final concentrations approximately 20 µM).

### Size exclusion chromatography (SEC)

SEC measurements were performed on a Superose 6 increase 10/300 column, a Superdex 75 PC 10/300 column or a Superdex 200 10/300 column (GE Healthcare). Analytical and preparative SEC measurements were executed on a Shimadzu LC-2010AHT HPLC and Agilent 1260 bio-inert HPLC, respectively. Samples (approximately 100 µg) were separated on the column with a flow rate of 0.5 mL/min.

### Transmission electron microscopy (TEM)

TEM grids (FCF-200-Cu, EMS) were glow-discharged using a Cressington carbon coater and power unit. Protein samples (0.2 mg/mL, 5 µL) were applied on the glow-discharged grids and incubated for 1 min. The samples were carefully removed using a filter paper and the grid was allowed to dry for at least 15 minutes. Then the grid was negatively stained by applying 2% uranyl acetate in water (5 µL). The staining solution was removed after 15 seconds and the grid was allowed to dry for at least 15 minutes. The samples were analyzed on a JEOL JEM-1010 TEM.

### Dynamic light scattering (DLS) measurements

DLS measurements were performed on a Zetasizer Nano S at 20 °C. Samples (20 µM) were centrifuged prior to analysis. Buffers were filtered prior to use. All measurements were done in triplo.

### Expression of His_6_-GFP

The pLEICS-05-GFP-H_6_ vector encoding for the hexahistidine-tagged GFP protein was previously constructed by the group of Geerten W. Vuister (Department of Biochemistry, University of Leicester) and kindly donated to our group. For a typical expression, LB medium (50 mL), containing ampicillin (100 mg/L) and chloramphenicol (50 mg/L), was inoculated with a single colony of *E*. *coli* BLR(DE3)pLysS containing pLEICS-05-GFP-H_6_ and was incubated overnight at 37 °C. This overnight culture was used to inoculate 2xTY medium (1 L), supplemented with ampicillin (100 mg/L) and chloramphenicol (50 mg/L). The culture was grown at 37 °C and protein expression was induced during logarithmic growth (OD_600_ = 0.4–0.6) by addition of IPTG (1 mM). After 5–6 h of expression at 30 °C, the cells were harvested by centrifugation (2700 g, 15 min, 4 °C) and the pellets were stored overnight at −20 °C.

After thawing, the cell pellet was resuspended in lysis buffer (50 mM NaH_2_PO_4_, 0.5 M NaCl, 10 mM imidazole, pH 8.0; 25 mL). The cells were lysed by ultrasonic disruption (5 times 30 s, 100% duty cycle, output control 3, Branson Sonifier 250, Marius Instruments). Then the lysate was centrifuged (16.400 g, 15 min, 4 °C) to remove the cellular debris. The supernatant was incubated with Ni-NTA agarose beads (3 mL) for 1 h at 4 °C. The suspension was loaded onto a column, the flow-through was collected and the beads were washed twice with wash buffer (50 mM NaH_2_PO_4_, 0.5 M NaCl, 20 mM imidazole, pH 8.0; 20 mL). Then, the protein of interest was eluted from the column with elution buffer (50 mM NaH_2_PO_4_, 0.5 M NaCl, 250 mM imidazole, pH 8.0; 1 time 0.5 mL, 7 times 1.5 mL). The elution fractions were analyzed by SDS-PAGE. The fractions containing His_6_-GFP were combined and concentrated (Amicon^®^ Ultra-15 Centrifugal Filter Device 10.000 NMWL). Further purification was performed by preparative SEC using a Superdex 200 10/300 column and PBS buffer as the eluent. The pure protein was obtained with a yield of 7.5 mg/L of culture. The purity was verified by SDS-PAGE and SEC using a Superose 6 10/300 GL column with PBS buffer as the eluent. ESI-TOF: calculated 29321.9 Da, found 29320.1 Da.

### Expression of His_6_-ELP-CCMV

The pET-15b-G-H_6_-[V_4_L_4_G_1_-9]-CCMV(ΔN26) vector encoding for the hexahistidine-tagged ELP-CCMV protein was previously constructed as described by van Eldijk *et al*.^19^. For a typical expression, LB medium (50 mL), containing ampicillin (100 mg/L) and chloramphenicol (50 mg/L), was inoculated with a single colony of *E. coli* BLR(DE3)pLysS containing pET-15b-G-H_6_-[V_4_L_4_G_1_-9]-CCMV(ΔN26), and was incubated overnight at 37 °C. This overnight culture was used to inoculate 2xTY medium (1 L), supplemented with ampicillin (100 mg/L) and chloramphenicol (50 mg/L). The culture was grown at 37 °C and protein expression was induced during logarithmic growth (OD_600_ = 0.4–0.6) by addition of IPTG (1 mM). After 6 h of expression at 30 °C, the cells were harvested by centrifugation (2700 g, 15 min, 4 °C) and the pellets were stored overnight at −20 °C.

After thawing, the cell pellet was resuspended in lysis buffer (50 mM NaH_2_PO_4_, 1.3 M NaCl, 10 mM imidazole, pH 8.0; 25 mL). The cells were lysed by ultrasonic disruption (5 times 30 s, 100% duty cycle, output control 3, Branson Sonifier 250, Marius Instruments). The lysate was incubated with DNase (10 mg/L) and RNase A (5 mg/L) for 10 min at 4 °C. Then the lysate was centrifuged (16.400 g, 15 min, 4 °C) to remove the cellular debris. The supernatant was incubated with Ni-NTA agarose beads (3 mL) for 1 h at 4 °C. The suspension was loaded onto a column, the flow-through was collected and the beads were washed twice with wash buffer (50 mM NaH_2_PO_4_, 1.3 M NaCl, 20 mM imidazole, pH 8.0; 20 mL). Then, the protein of interest was eluted from the column with elution buffer (50 mM NaH_2_PO_4_, 1.3 M NaCl, 250 mM imidazole, pH 8.0; 1 time 0.5 mL, 7 times 1.5 mL). The elution fractions were analyzed by SDS-PAGE. The fractions containing His_6_-ELP-CCMV were combined and dialyzed against dimer buffer to obtain the protein dimers. For storage the proteins were assembled by dialysis against pH-induced assembly buffer. The pure protein was obtained with a yield of 100 mg/L of culture. The purity was verified by SDS-PAGE. The geometry and assembly properties were analyzed by SEC using a Superose 6 increase 10/300 column with pH-induced assembly buffer as the eluent and TEM. ESI-TOF: calculated 22253.4 Da, found 22253.5 Da.

### Purification of wt-CCMV

The wild-type CCMV virus, which was extracted from the cowpea plant, was kindly donated to our group by the group of Jeroen J. L. M. Cornelissen (Biomolecular Nanotechnology Department, University of Twente). The virus (11 mg/mL, 500 µL) was dialyzed against RNA buffer (4 °C, first 2 h 200 mL, then overnight 800 mL). The precipitated RNA was centrifuged down (4 °C, 13.000 rpm, overnight). The supernatant was dialyzed against clean buffer (4 °C, two times 3 h 330 mL, then overnight 330 mL). The proteins were assembled into capsid by dialysis against storage buffer (4 °C, three times 3 h 330 mL), after which the pure wt CCMV capsid protein was obtained (8.67 mg/mL). The purity was verified by SDS-PAGE. The geometry and assembly properties were analyzed by SEC using a Superose 6 increase 10/300 column with pH-induced assembly buffer as the eluent. ESI-TOF: calculated 20254.3 Da (with acetylation), found 20254.5 Da.

### Expression of His_6_-PAMO

The vector encoding for the hexahistidine-tagged fusion protein His_6_-BVMO-PAMO was previously constructed by the group of Marco W. Fraaije (Department of Biotechnology, University of Groningen) and kindly donated to our group^21^. For a typical expression, LB medium (50 mL), containing ampicillin (10 mg/L), was inoculated with a single colony of *E. coli* TOP10 containing the vector encoding His_6_-BVMO-PAMO and was incubated overnight at 37 °C. This overnight culture was used to inoculate TB medium (1 L), supplemented with ampicillin (50 mg/L), _L_-arabinose (0.02% v.v^−1^) and glucose (0.05% v.v^−1^). The culture was grown for 30 hours at 24 °C. The cells were harvested by centrifugation (2700 g, 15 min, 4 °C) and the pellets were stored overnight at −20 °C.

After thawing, the cell pellet was resuspended in lysis buffer (50 mM Tris·HCl, pH 7.5; 25 mL). Flavin adenine dinucleotide (FAD) (100 mM stock solution, 25 µL) was added to the resuspended cells. The cells were lysed by ultrasonic disruption (7 times 10 s, 100% duty cycle, output control 3, Branson Sonifier 250, Marius Instruments). Then the lysate was centrifuged (16.400 g, 15 min, 4 °C) to remove the cellular debris. The supernatant was incubated with Ni-NTA agarose beads (3 mL) for 1 h at 4 °C. The suspension was loaded onto a column, the flow-through was collected and the beads were washed twice with wash buffer (50 mM Tris·HCl, 5 mM imidazole, pH 7.5; 20 mL). Then, the protein of interest was eluted from the column with elution buffer (50 mM Tris·HCl, 500 mM imidazole, pH 7.5; 7 times 1.0 mL). The elution fractions were analyzed by SDS-PAGE. The fractions containing His_6_-PAMO were combined and dialyzed against PAMO buffer (Amicon^®^ Ultra-15 Centrifugal Filter Device 10.000 NMWL). The pure protein was obtained with a yield of 16.7 mg/L of culture. The purity was verified by SDS-PAGE. ESI-TOF: calculated 100235.3 Da, found 100230.0 Da.

### Expression of His_6_-CalB

The pET-22b-His_6_-CalB vector encoding for bacterial expression of the histidine-tagged wild type CalB protein was previously constructed as described by Schoffelen *et al* ^23^. For a typical expression, LB medium (50 mL), containing ampicillin (100 mg/L) and chloramphenicol (50 mg/L), was inoculated with a single colony of *E. coli* B834(DE3)pLysS containing pET-22b-His_6_-CalB and was incubated overnight at 37 °C. This overnight culture was used to inoculate 2xTY medium (1 L), supplemented with ampicillin (100 mg/L) and chloramphenicol (50 mg/L). The culture was grown at 37 °C and protein expression was induced during logarithmic growth (OD_600_ = 0.4–0.6) by addition of IPTG (1 mM). After 25 h of expression at 25 °C, the cells were harvested by centrifugation (2700 g, 15 min, 4 °C) and the pellets were stored overnight at −20 °C.

After thawing, the cell pellet was resuspended in lysis buffer (50 mM NaH_2_PO_4_, 0.5 M NaCl, 10 mM imidazole, pH 8.0; 25 mL). The cells were lysed by ultrasonic disruption (5 times 30 s, 100% duty cycle, output control 3, Branson Sonifier 250, Marius Instruments). Then the lysate was centrifuged (16.400 g, 15 min, 4 °C) to remove the cellular debris. The supernatant was incubated with Ni-NTA agarose beads (3 mL) for 1 h at 4 °C. The suspension was loaded onto a column, the flow-through was collected and the beads were washed twice with wash buffer (50 mM NaH_2_PO_4_, 0.5 M NaCl, 20 mM imidazole, pH 8.0; 20 mL). Then, the protein of interest was eluted from the column with elution buffer (50 mM NaH_2_PO_4_, 0.5 M NaCl, 250 mM imidazole, pH 8.0; 1 time 0.5 mL, 7 times 1.5 mL). The elution fractions were analyzed by SDS-PAGE. The fractions containing His_6_-CalB were combined and concentrated (Amicon^®^ Ultra-4 Centrifugal Filter Device 10.000 NMWL). Further purification was performed by preparative SEC using a Superdex 75 PC 10/300 column and CalB buffer as the eluent. The pure protein was obtained with a yield of 1.2–1.9 mg/L of culture. The purity was verified by SDS-PAGE and SEC using a Superose 6 10/300 GL column and CalB buffer as the eluent. ESI-TOF: calculated 34269.7 Da, found 34268.0 Da.

### General protocol spectrophotometric activity assays

Esterase activity was analyzed by the hydrolysis of *p*-nitrophenol acetate (*p*-NPA). The catalyst (101.0 µM in buffer, 99.0 µL) was added to *p*-NPA (100 mM in DMSO, 1.0 µL) in triplo. The background hydrolysis was measured by the addition of the desired buffer without catalyst to the substrate. The production of *p*-nitrophenol (*p*-NP) was monitored for 10 minutes at 21 °C by measuring the absorbance at 405 nm on a Tecan Spark 10 M microplate reader. The slopes of the curves were taken as a measure of the hydrolytic activity. Calibration curves of the absorbance of *p*-nitrophenol in the different buffers were measured, in order to convert the measured absorbance to µmol/min.

### Data availability

All relevant data generated or analyzed during this study are included in this published article (and its Supplementary Information files).

## Electronic supplementary material

supplementary information
