# Supplementary material for: Alternative application of an affinity purification tag: hexahistidines in ester hydrolysis
Source: Sci Rep. 2017 Nov 7;7:14772. doi: 10.1038/s41598-017-15310-y (PMC5676709; doi:10.1038/s41598-017-15310-y)
Supplement: Supplementary file 1 — supplementary information [file 41598_2017_15310_MOESM1_ESM.doc]

Supporting Information

Alternative application of an affinity purification tag: hexahistidines in ester hydrolysis

*Lise Schoonen,^b^ Kayleigh S. van Esterik,^b^ Chunqiu Zhang,^c^ Rein V. Ulijn,^c^ Roeland R. M. Nolte^b^ and Jan C. M. van Hest^a,b^**

a. Eindhoven University of Technology, PO Box 513 (STO 3.31), 5600 MB Eindhoven, The Netherlands.

b. Radboud University, Institute for Molecules and Materials, Heyendaalseweg 135, 6525 AJ Nijmegen, The Netherlands.

c. Advanced Science Research Center, City University of New York, 85 St Nicholas Terrace, New York 10031, USA.

List of contents

**1** Experimental section

|  | 1.1 | Expression of His_6_-GFP | 3 |
| --- | --- | --- | --- |
|  | 1.2 | Expression of His_6_-ELP-CCMV | 5 |
|  | 1.3 | Purification of wt CCMV capsid proteins | 7 |
|  | 1.4 | Expression of His_6_-PAMO | 9 |
|  | 1.5 | Expression of His_6_-CalB | 10 |
|  | 1.6 | Oligo and protein sequences | 12 |
|  | 1.7 | Spectrophotometric activity assays | 13 |

**2 Supplemental figures** 14

**3 References** 22

1 Experimental section

**1.1 Expression of His_6_-GFP**


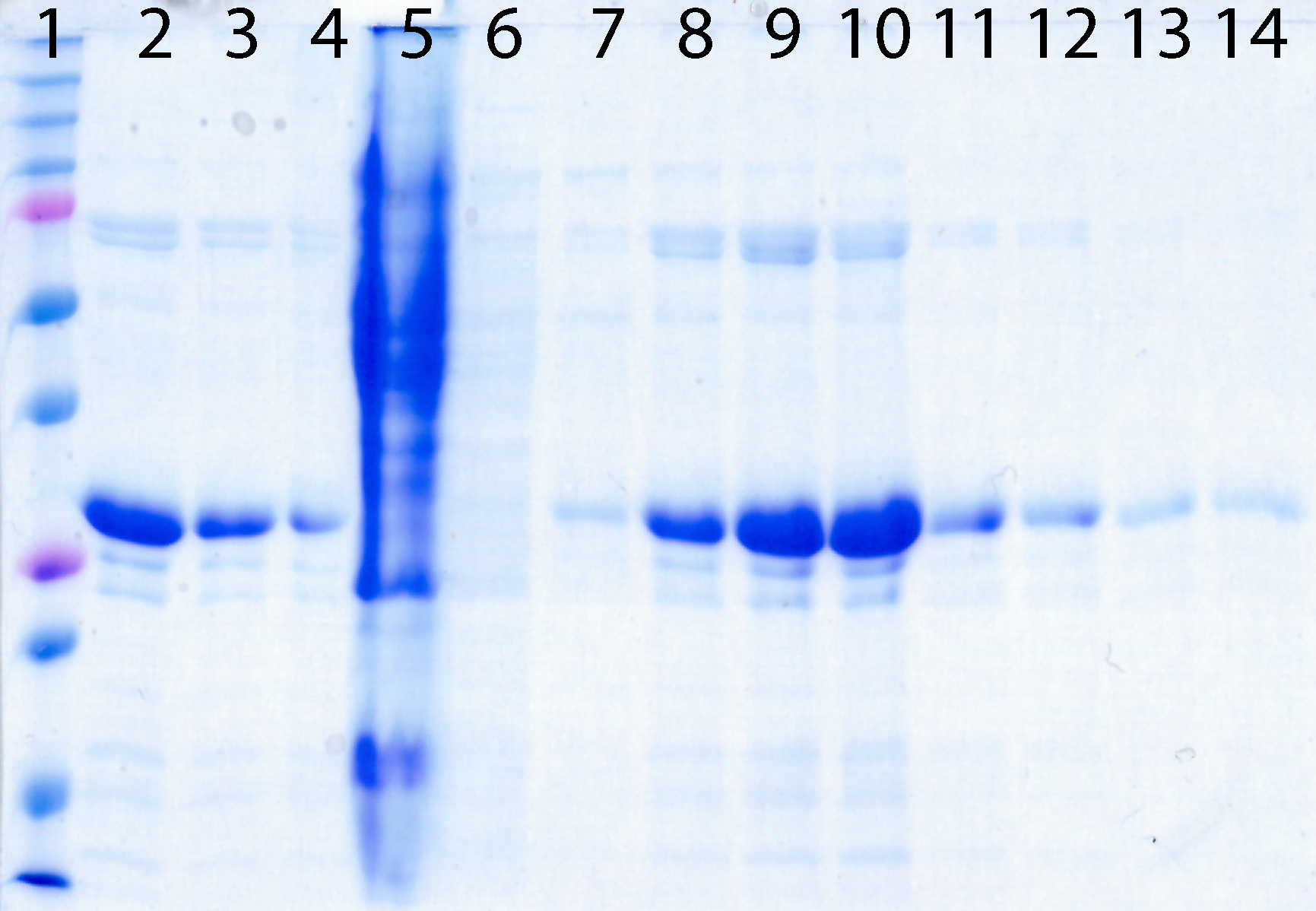

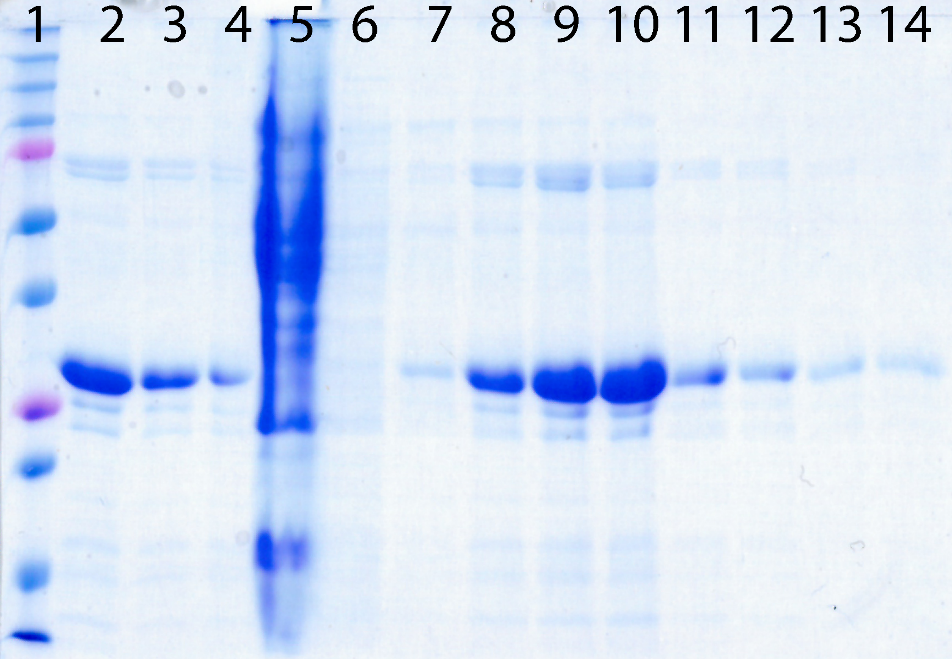

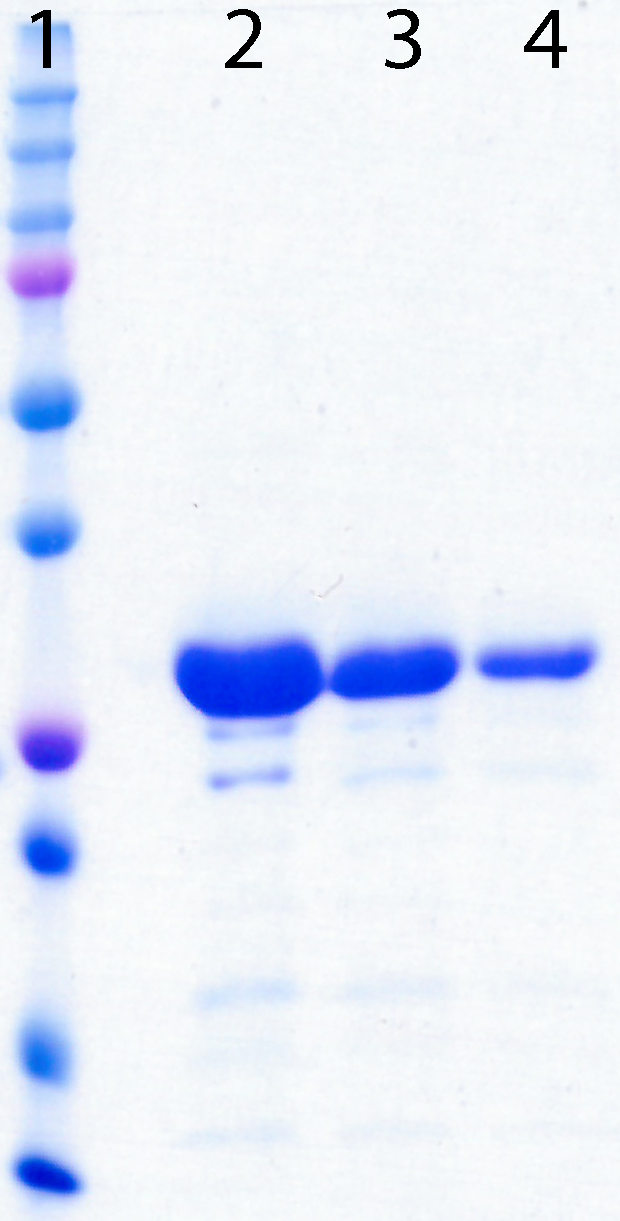

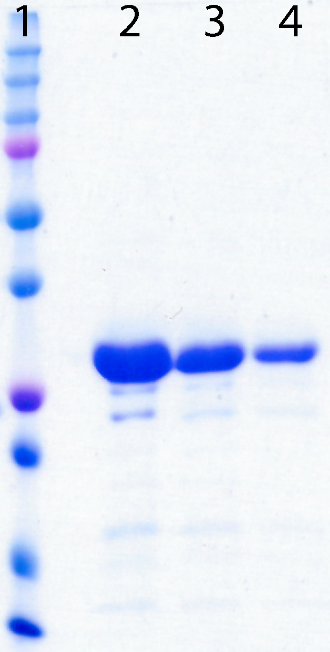


75 kDa -

50 kDa -

37 kDa -

25 kDa -

20 kDa -

75 kDa -

50 kDa -

37 kDa -

25 kDa -

20 kDa -

**SDS-PAGE analysis of affinity purification of His_6_-GFP (left) and purified His_6_-GFP (right).**


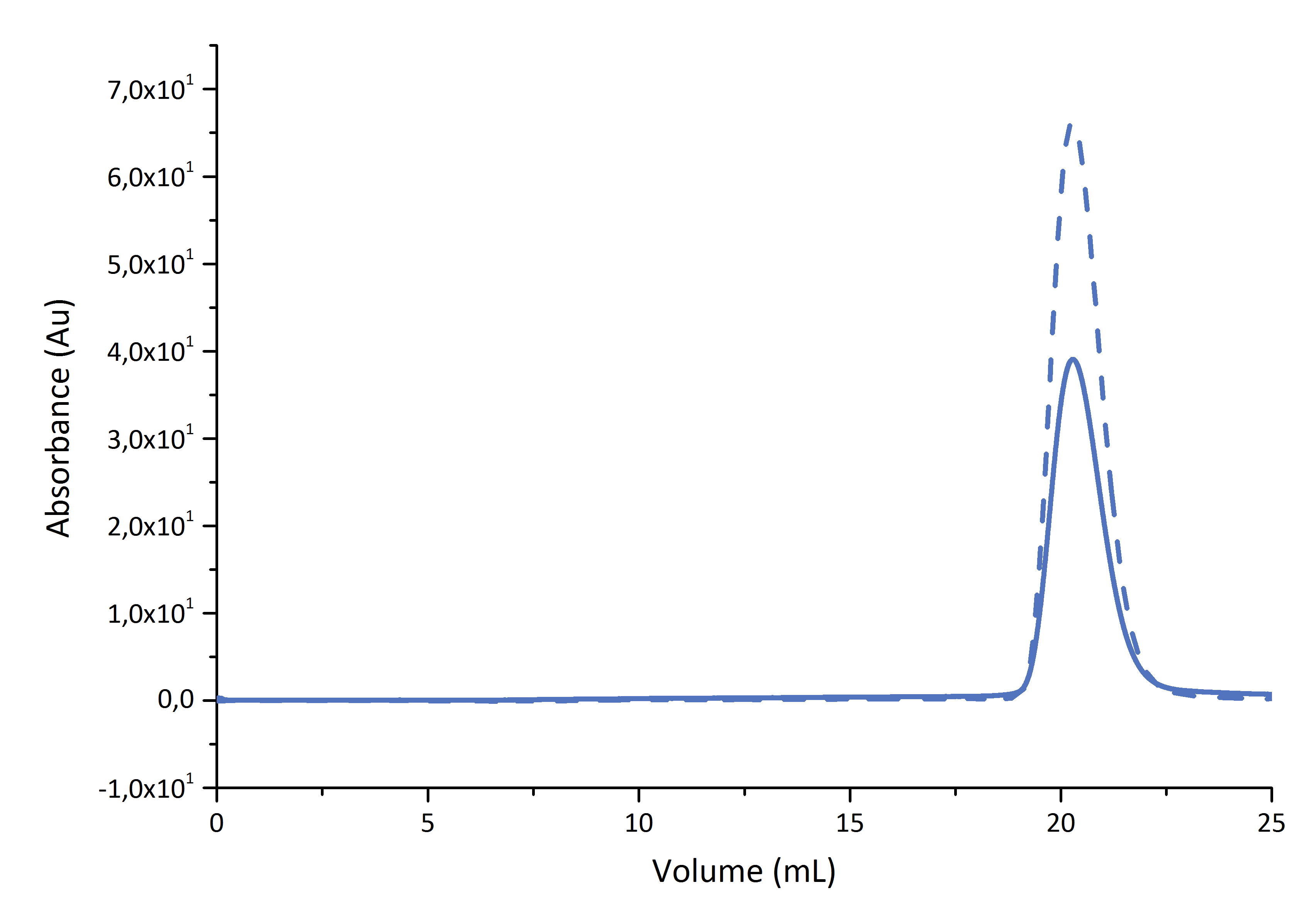


**Size exclusion chromatogram of purified His_6_-GFP in pH-induced assembly buffer.** Solid line = 280 nm, dashed line = 495 nm.


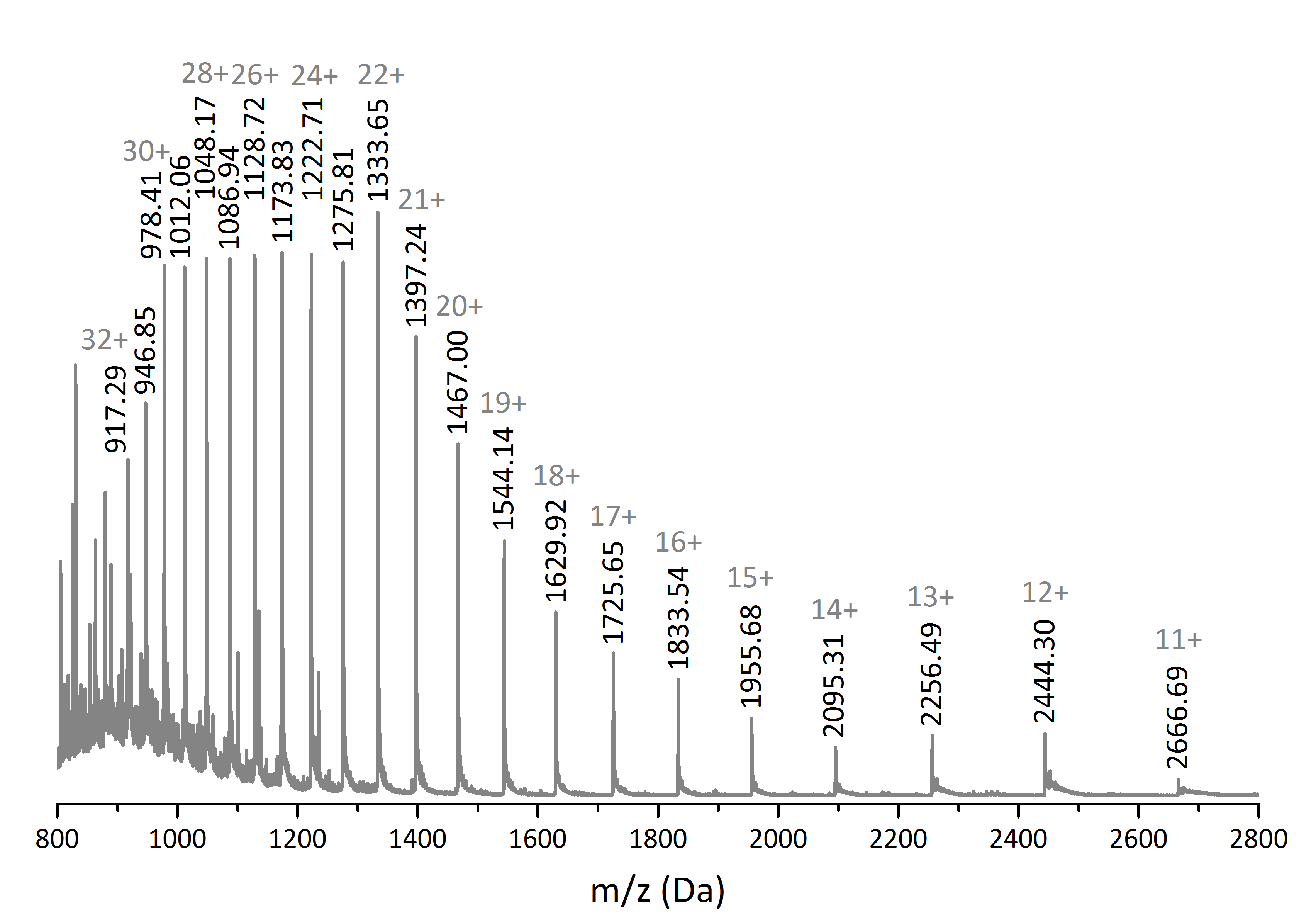

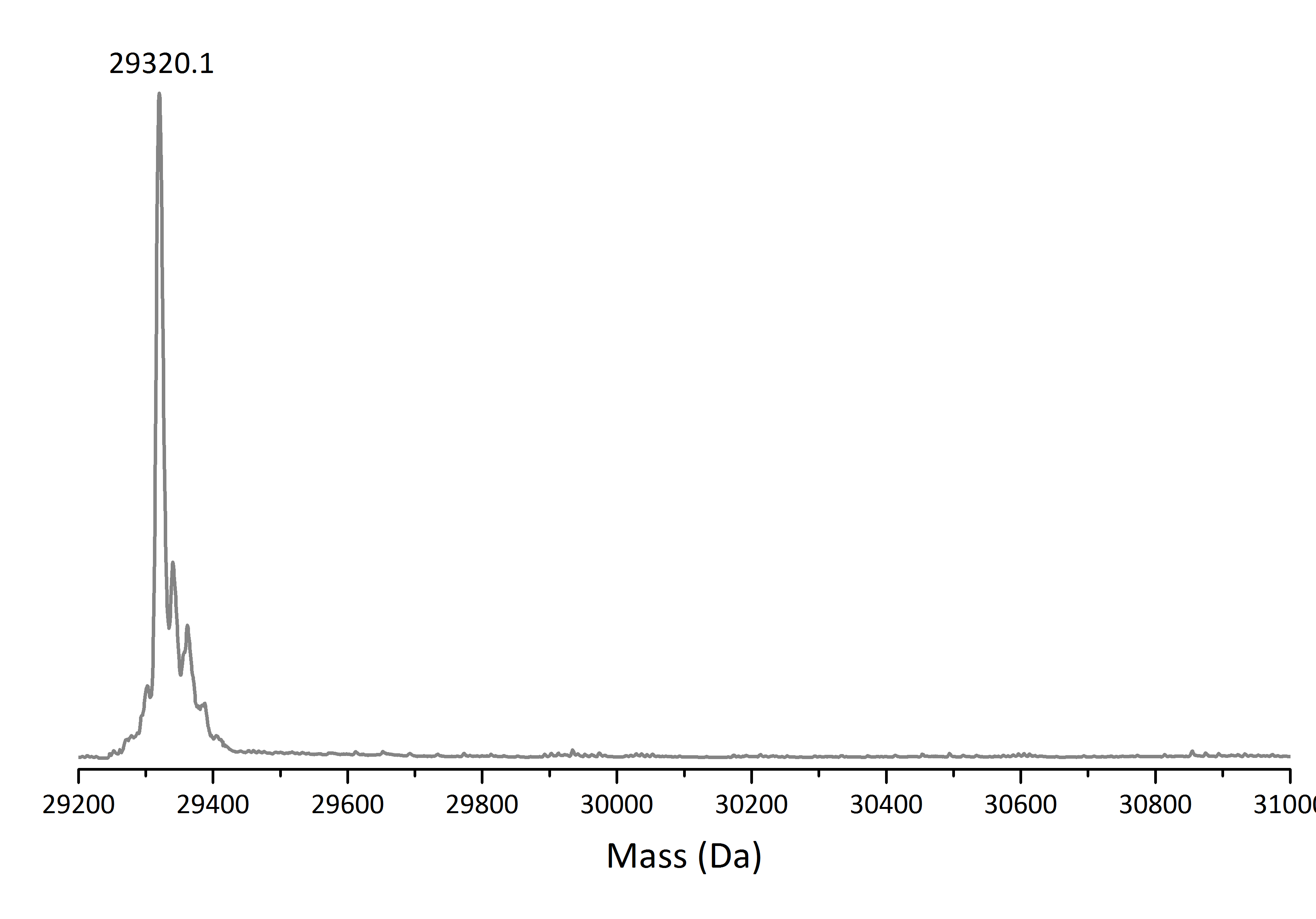


**ESI-TOF mass spectrometry of purified His_6_-GFP.** Deconvoluted total mass spectrum and multiply charged ion series (inset). The expected molecular weight is 29321.9 Da.

**1.2 Expression of His_6_-ELP-CCMV**


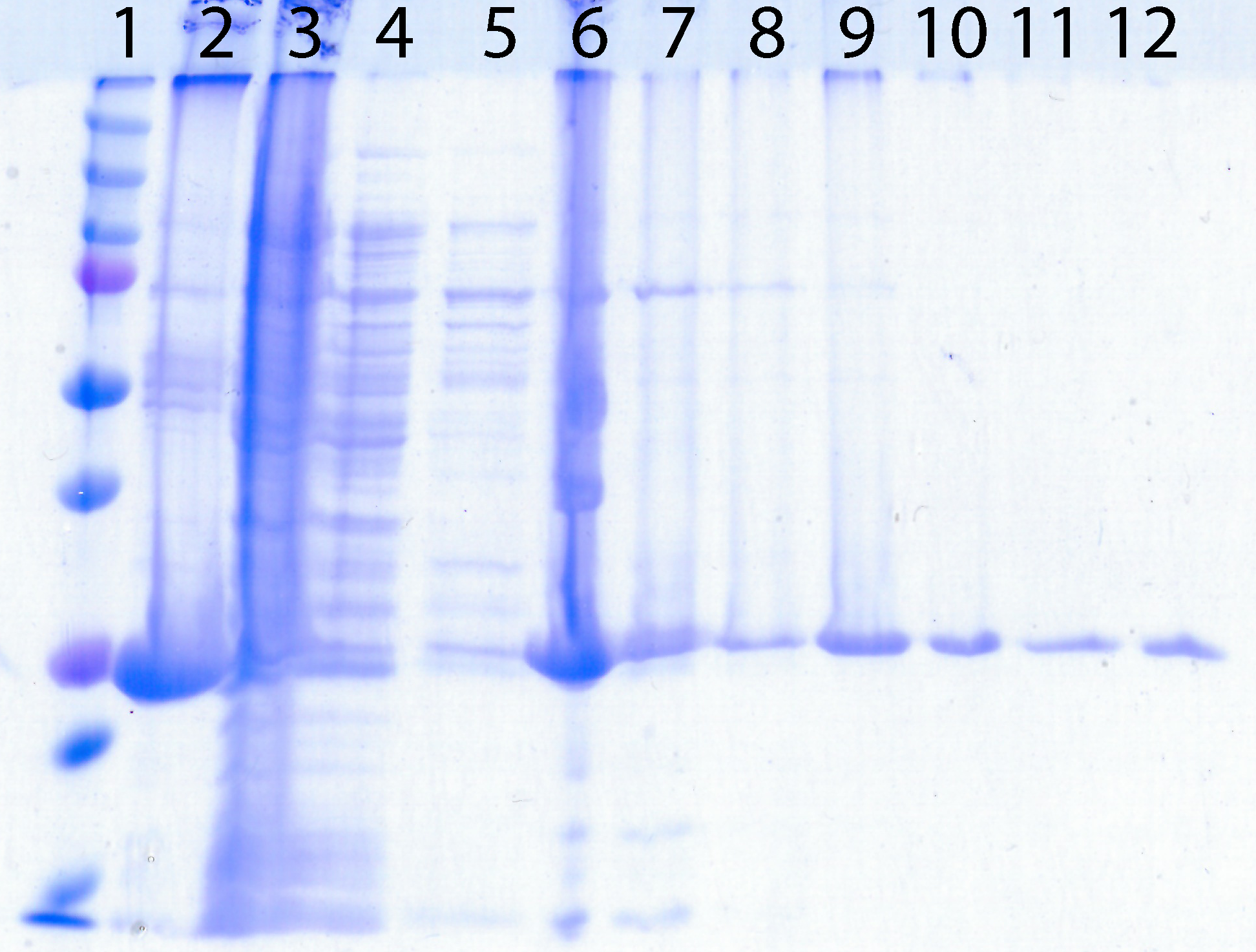

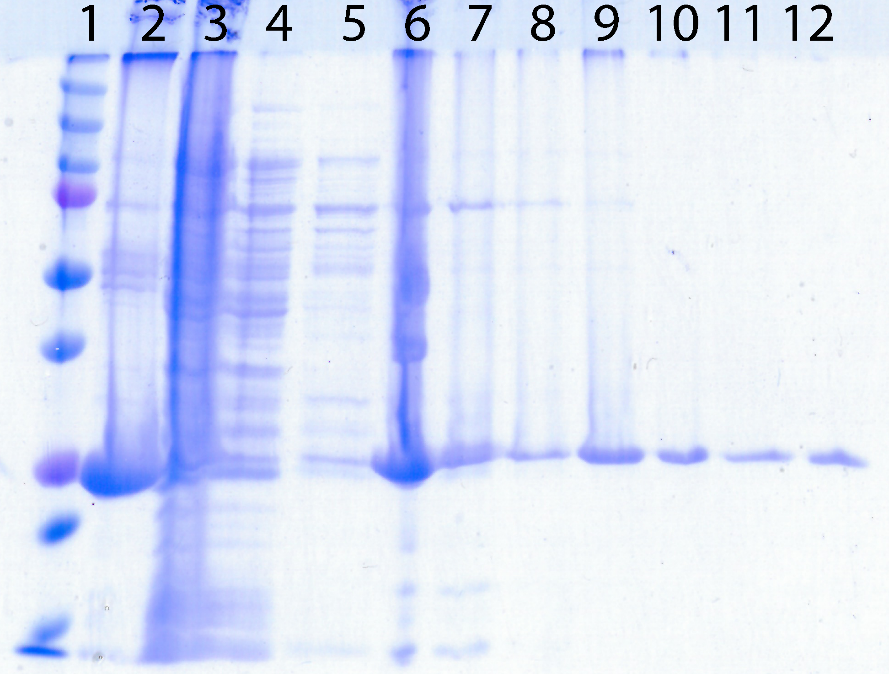

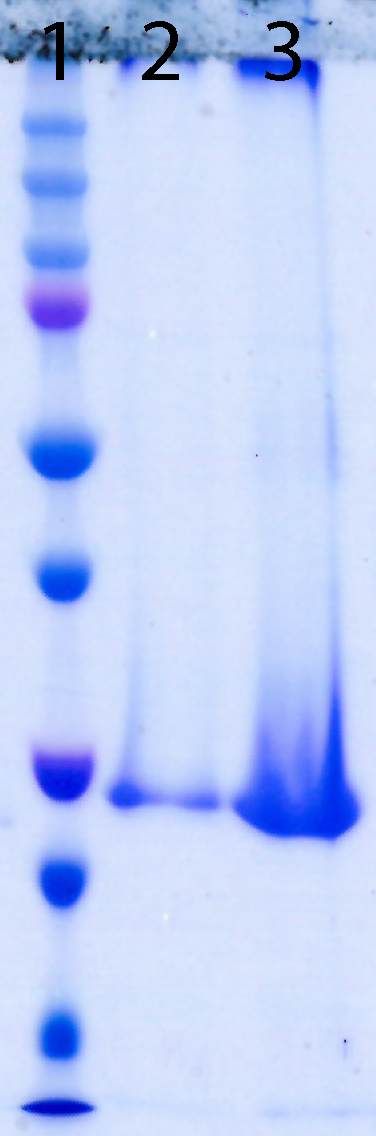


75 kDa -

50 kDa -

37 kDa -

25 kDa -

20 kDa -

75 kDa -

50 kDa -

37 kDa -

25 kDa -

20 kDa -

**SDS-PAGE analysis of affinity purification of His_6_-ELP-CCMV (left) and purified His_6_-ELP-CCMV (right).**


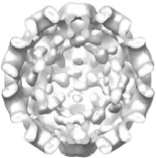


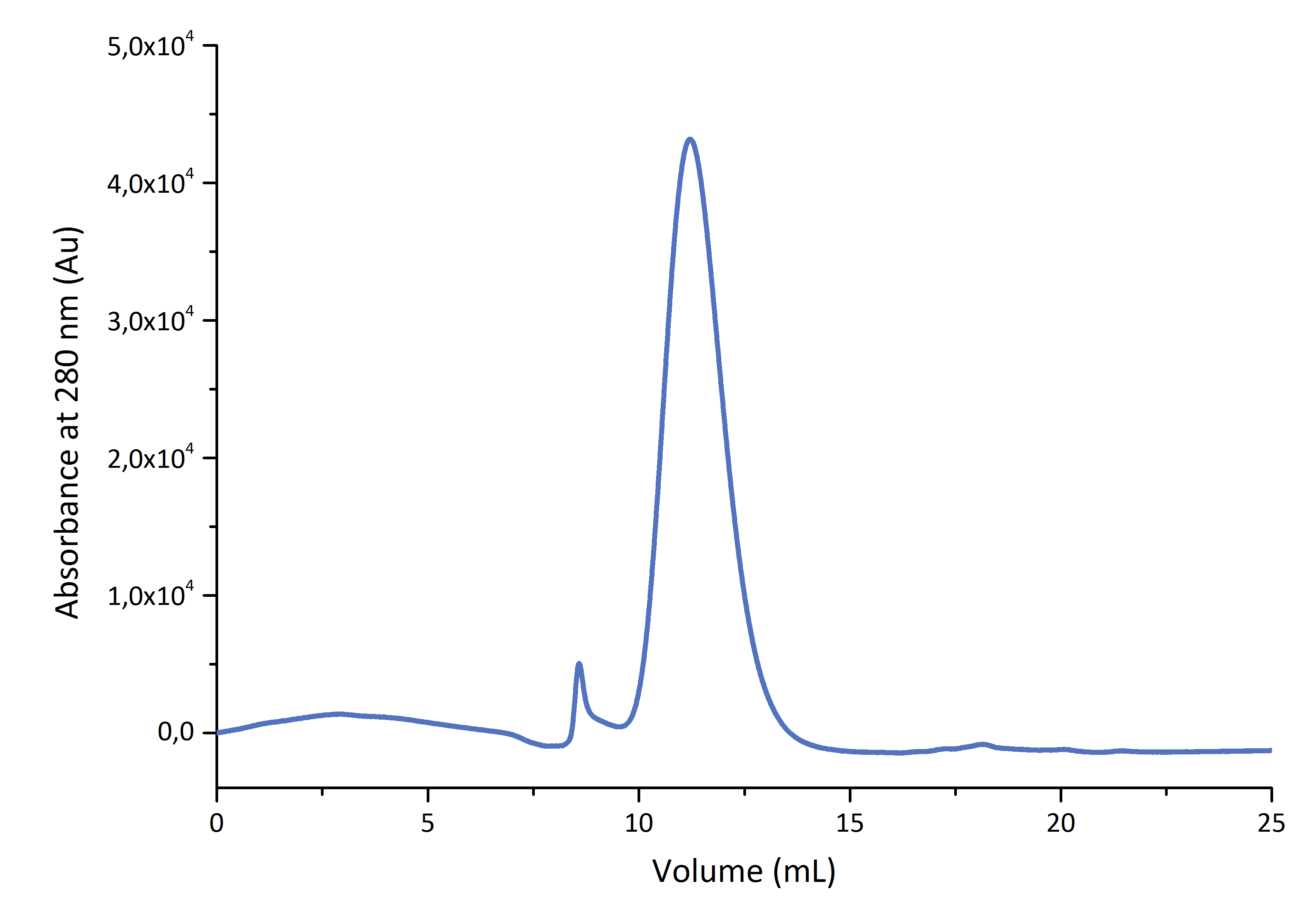


**Size exclusion chromatogram of purified His_6_-ELP-CCMV in pH-induced assembly buffer.**


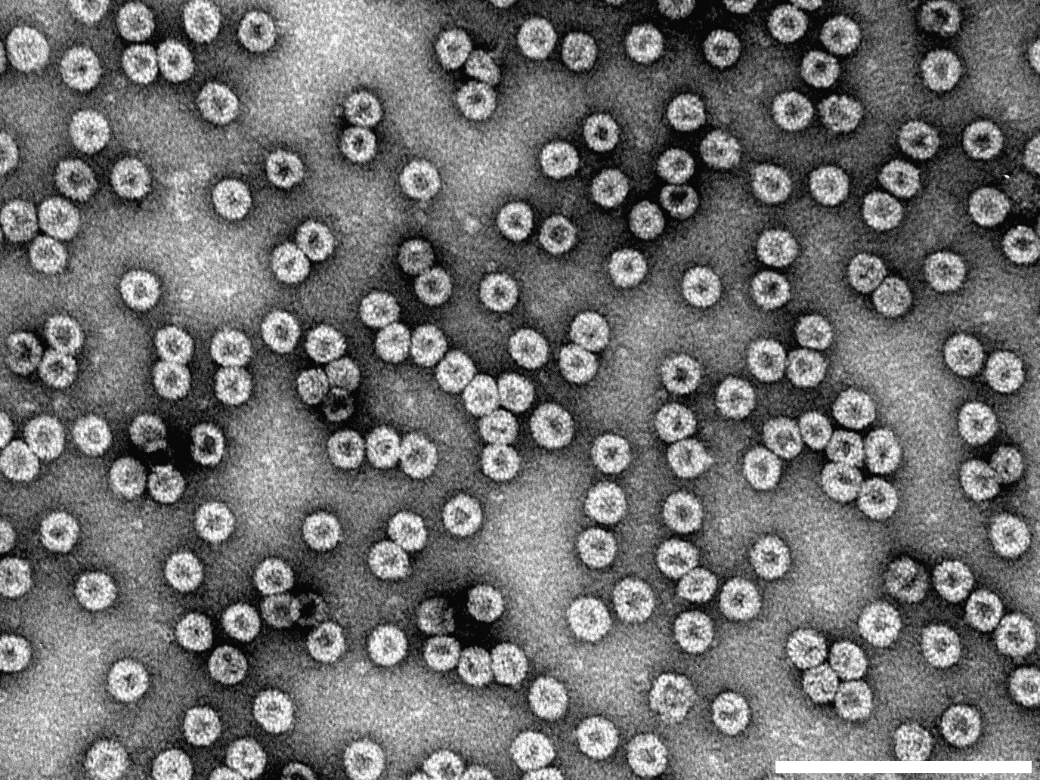


**Uranyl acetate stained TEM micrograph of His_6_-ELP-CCMV.** Average particle size = 29.2 ± 1.5 nm. Scale bar corresponds to 200 nm.


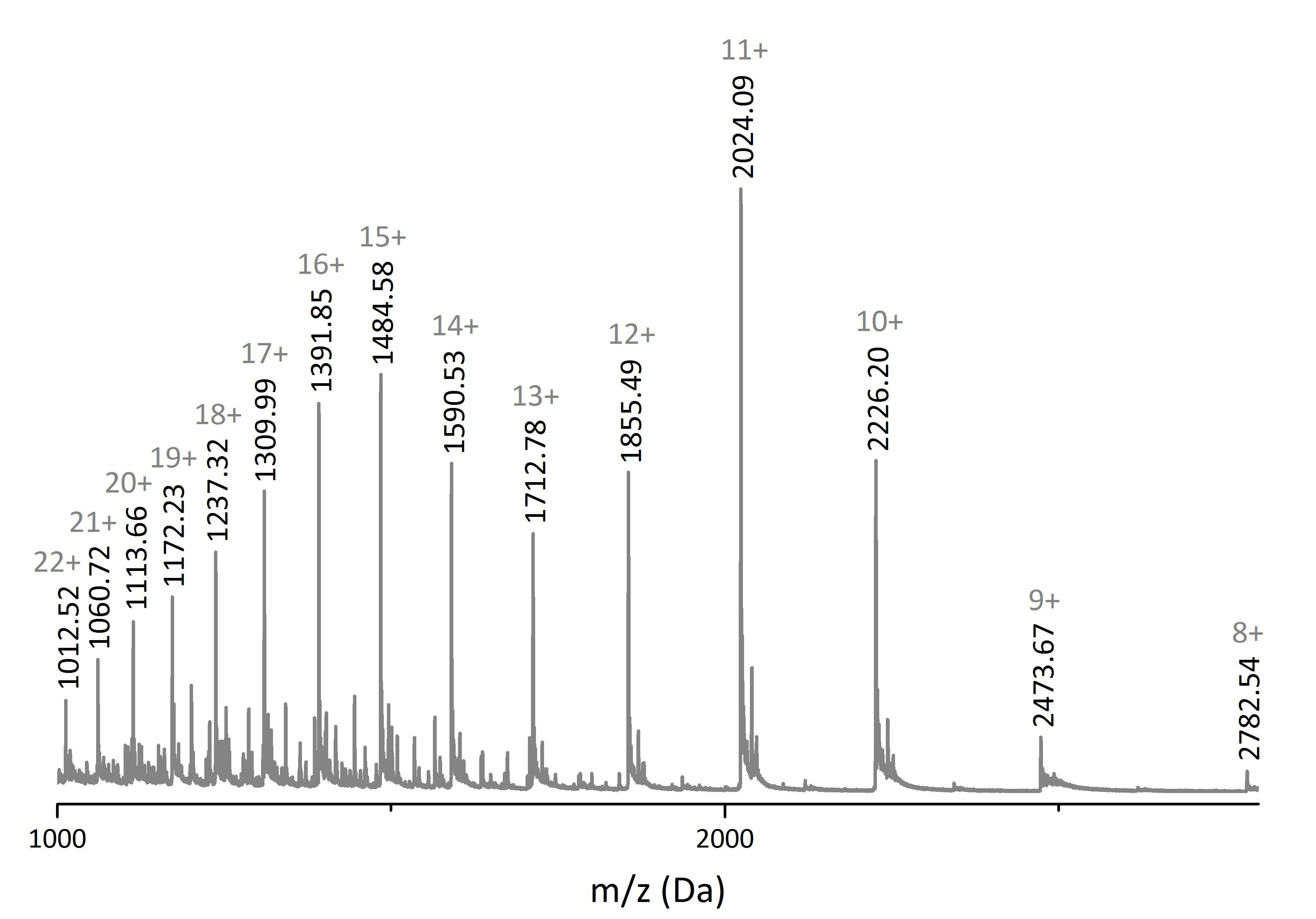

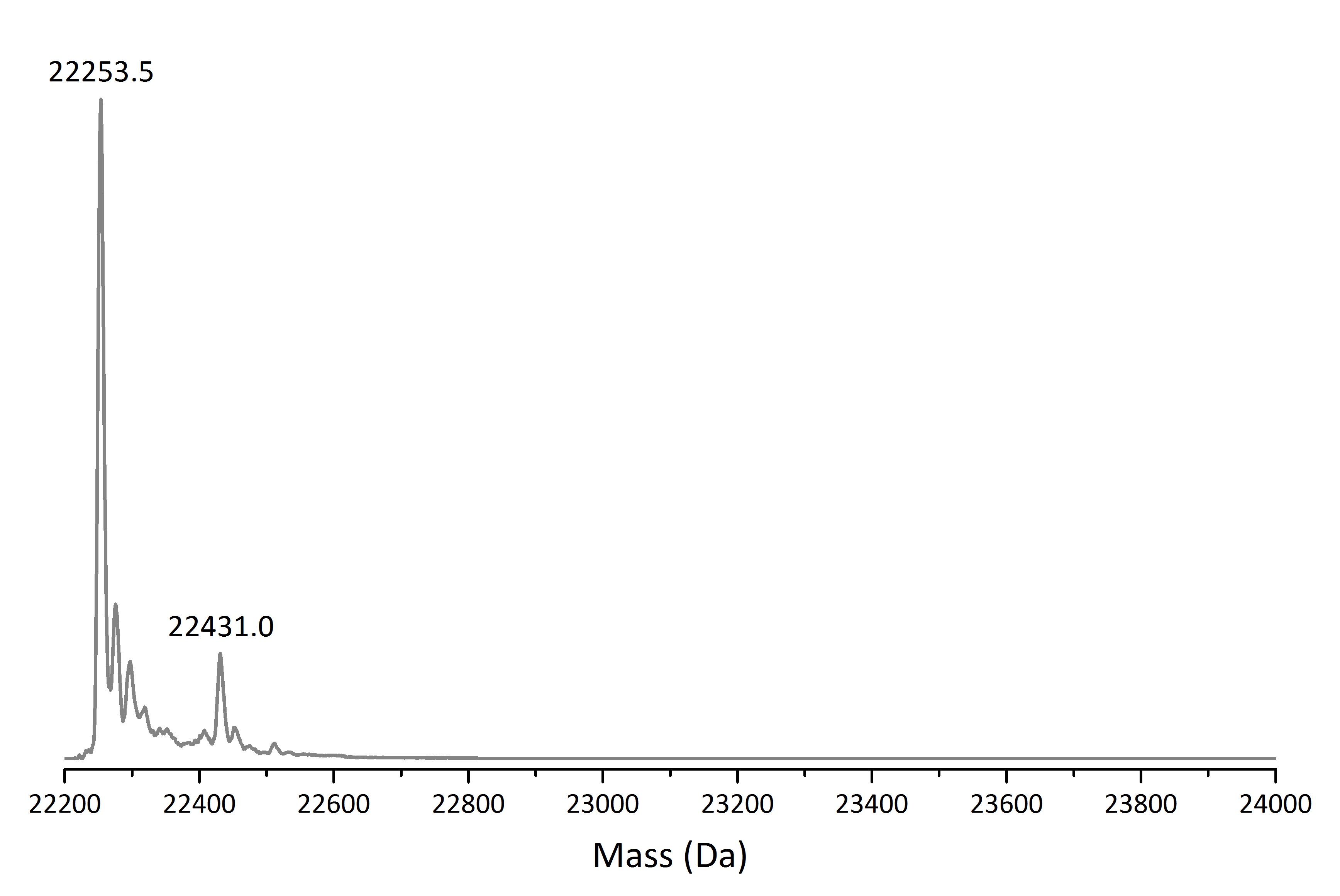


**ESI-TOF mass spectrometry of purified His_6_-ELP-CCMV.** Deconvoluted total mass spectrum and multiply charged ion series (inset). The expected molecular weight is 22253.4 Da.

**1.3 Purification of wt CCMV**


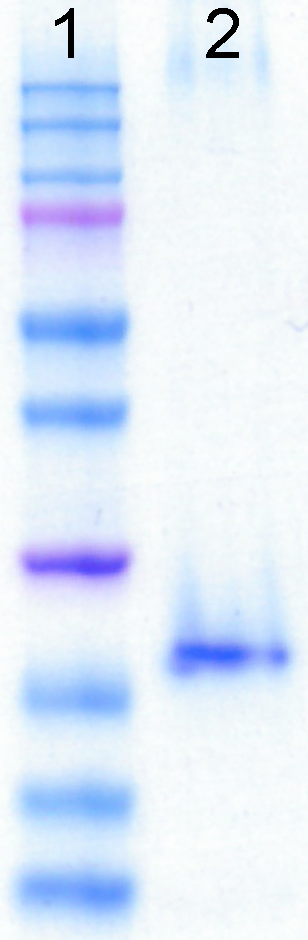


75 kDa -

50 kDa -

37 kDa -

25 kDa -

20 kDa -

**SDS-PAGE analysis of purified wt CCMV capsid protein.**


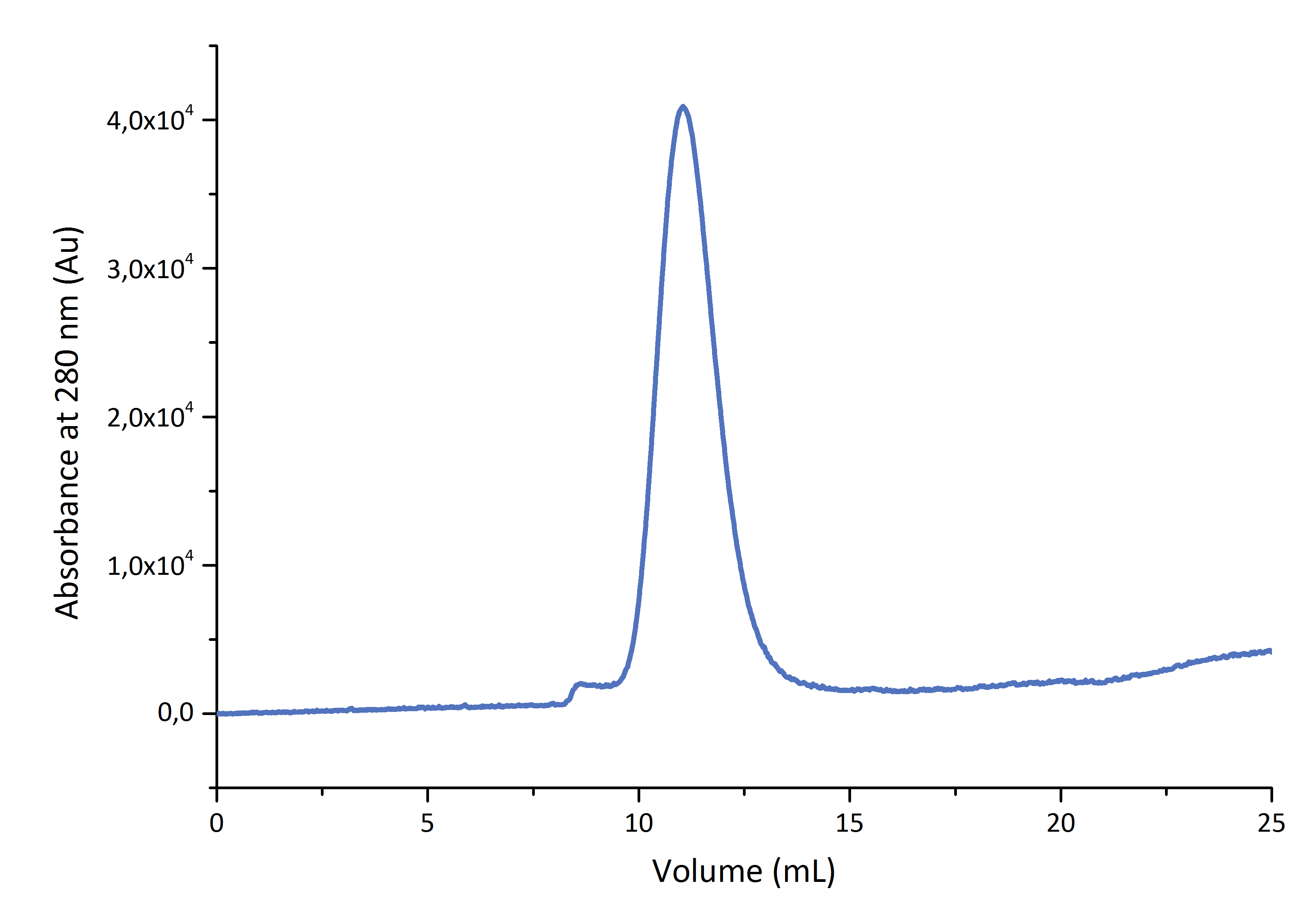


**Size exclusion chromatogram of purified wt CCMV capsid protein in pH-induced assembly buffer.**


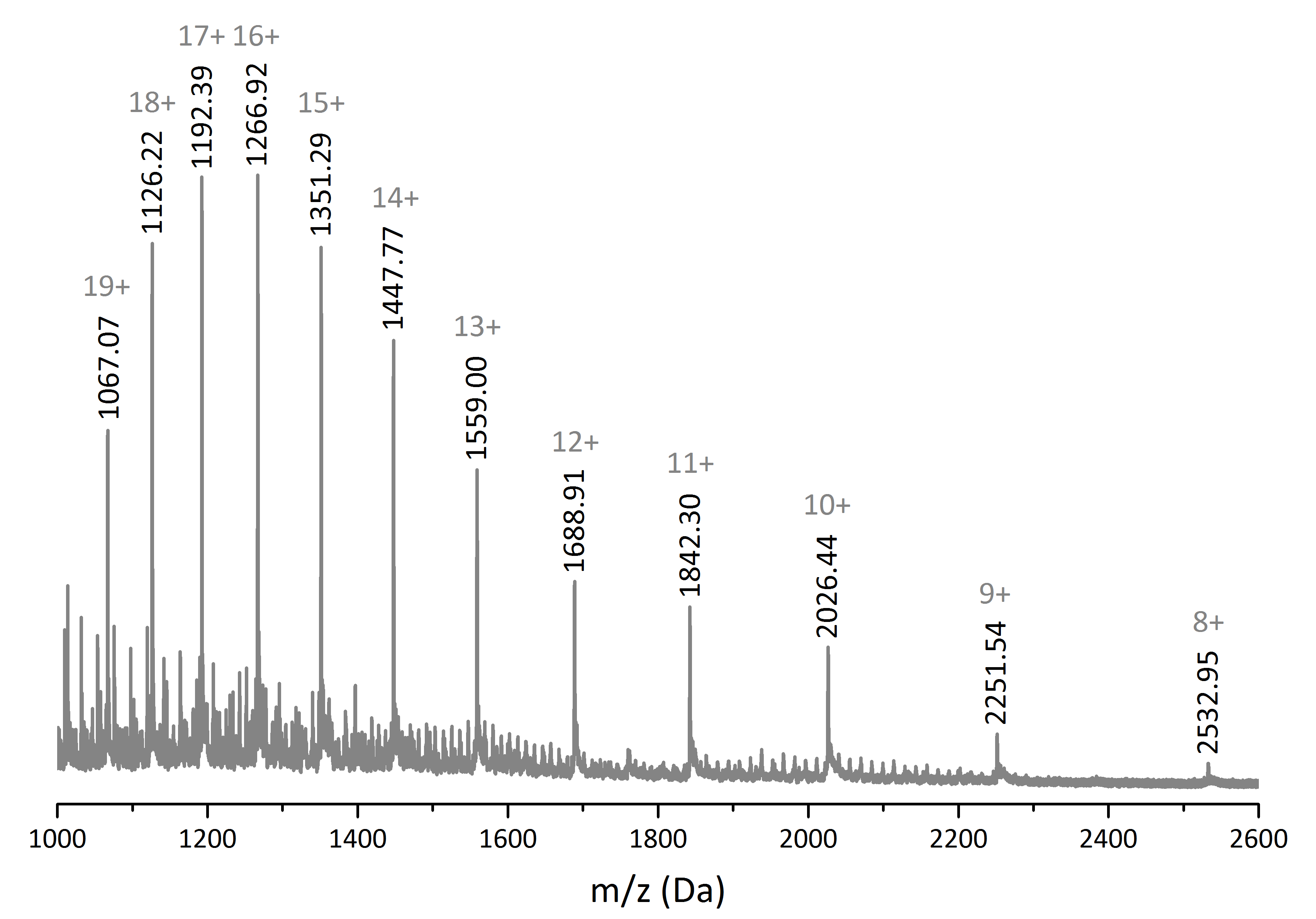

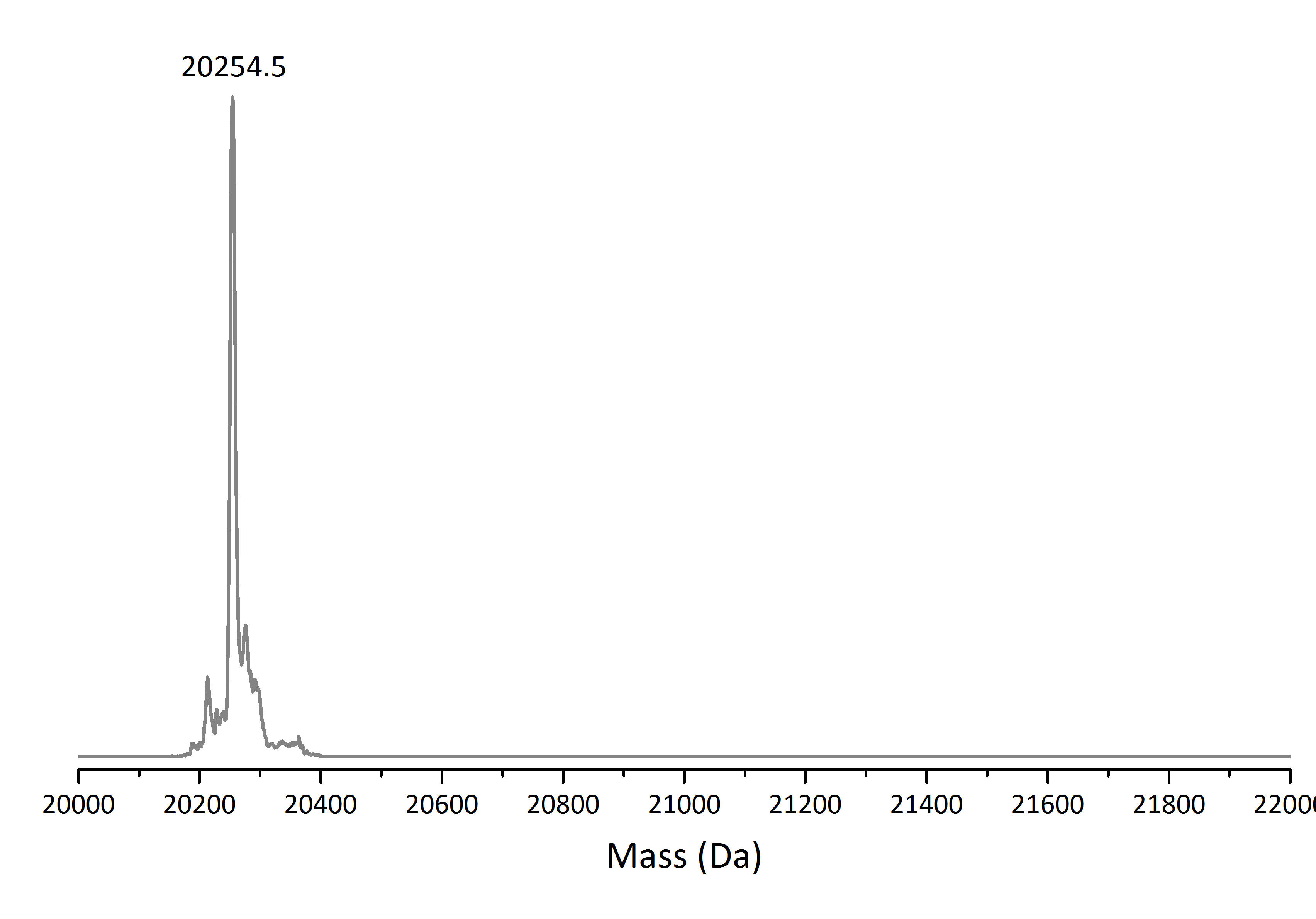


**ESI-TOF mass spectrometry of purified wt CCMV capsid protein.** Deconvoluted total mass spectrum and multiply charged ion series (inset). The expected molecular weight is 20254.3 Da.

**1.4 Expression of His_6_-PAMO**


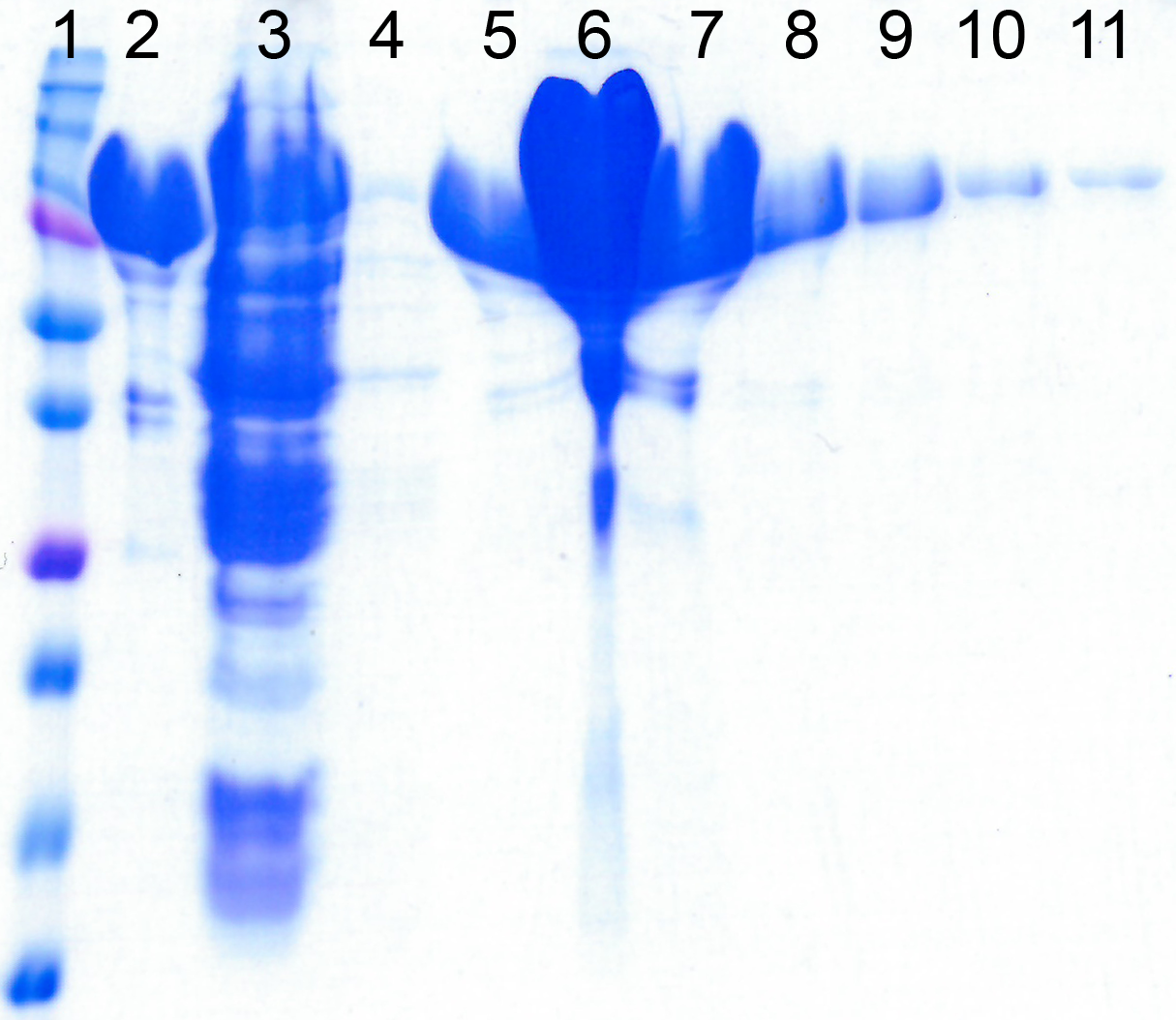


100 kDa -

75 kDa -

50 kDa -

37 kDa -

25 kDa -

20 kDa -

**SDS-PAGE analysis of affinity purification of His_6_-PAMO.**


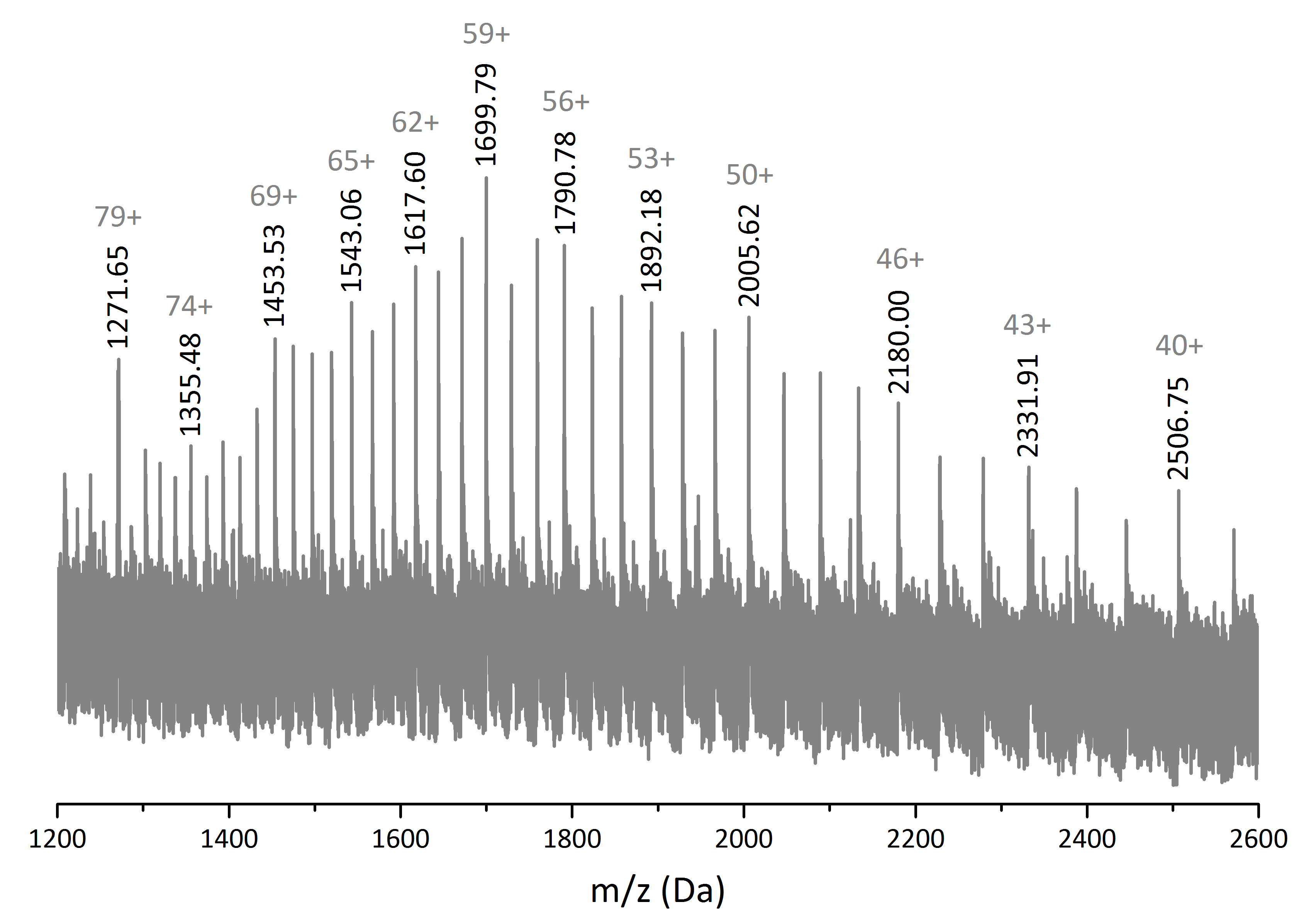


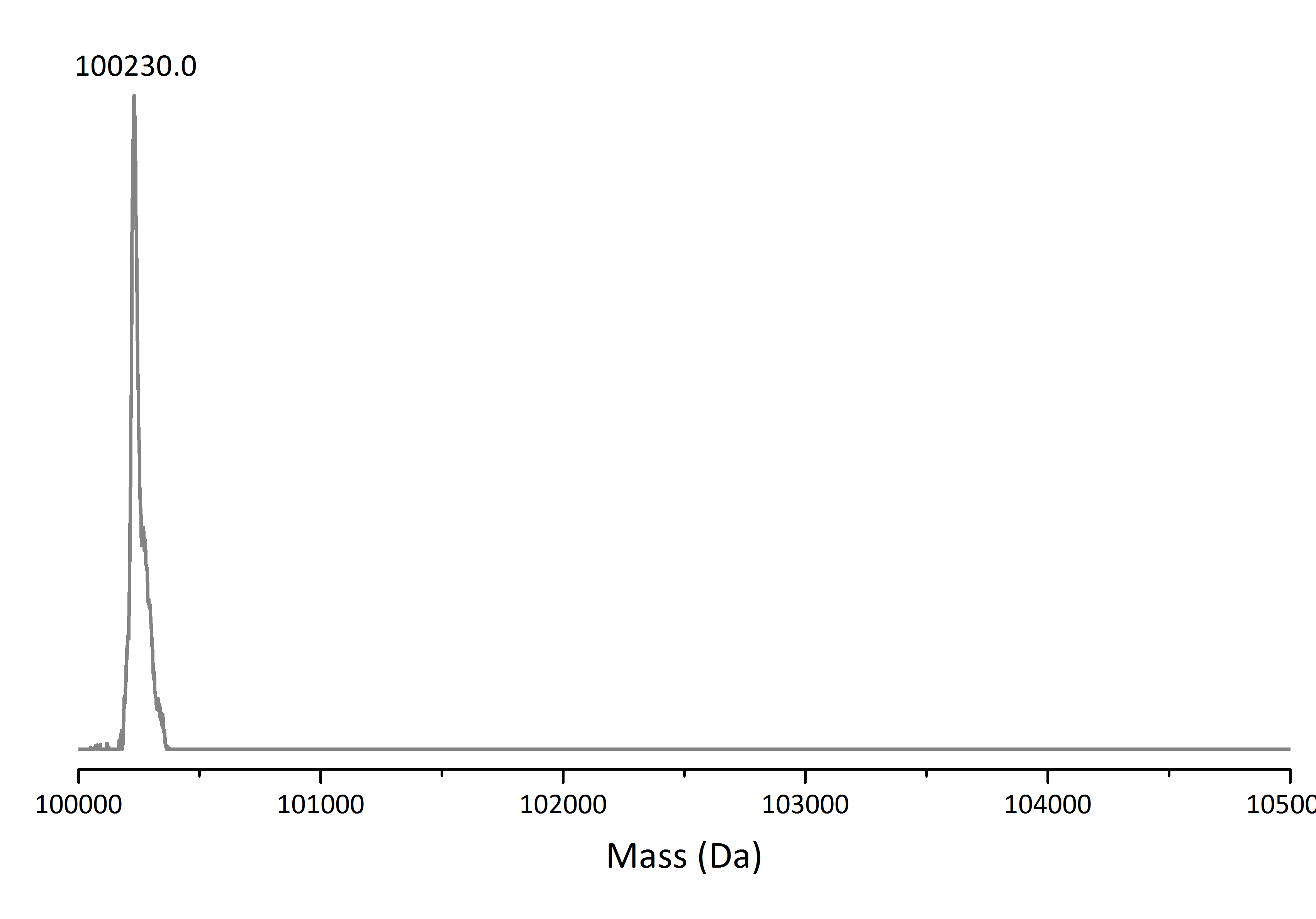


**ESI-TOF mass spectrometry of purified His_6_-PAMO.** Deconvoluted total mass spectrum and multiply charged ion series (inset). The expected molecular weight is 100235.3 Da.

**1.5 Expression of His_6_-CalB**


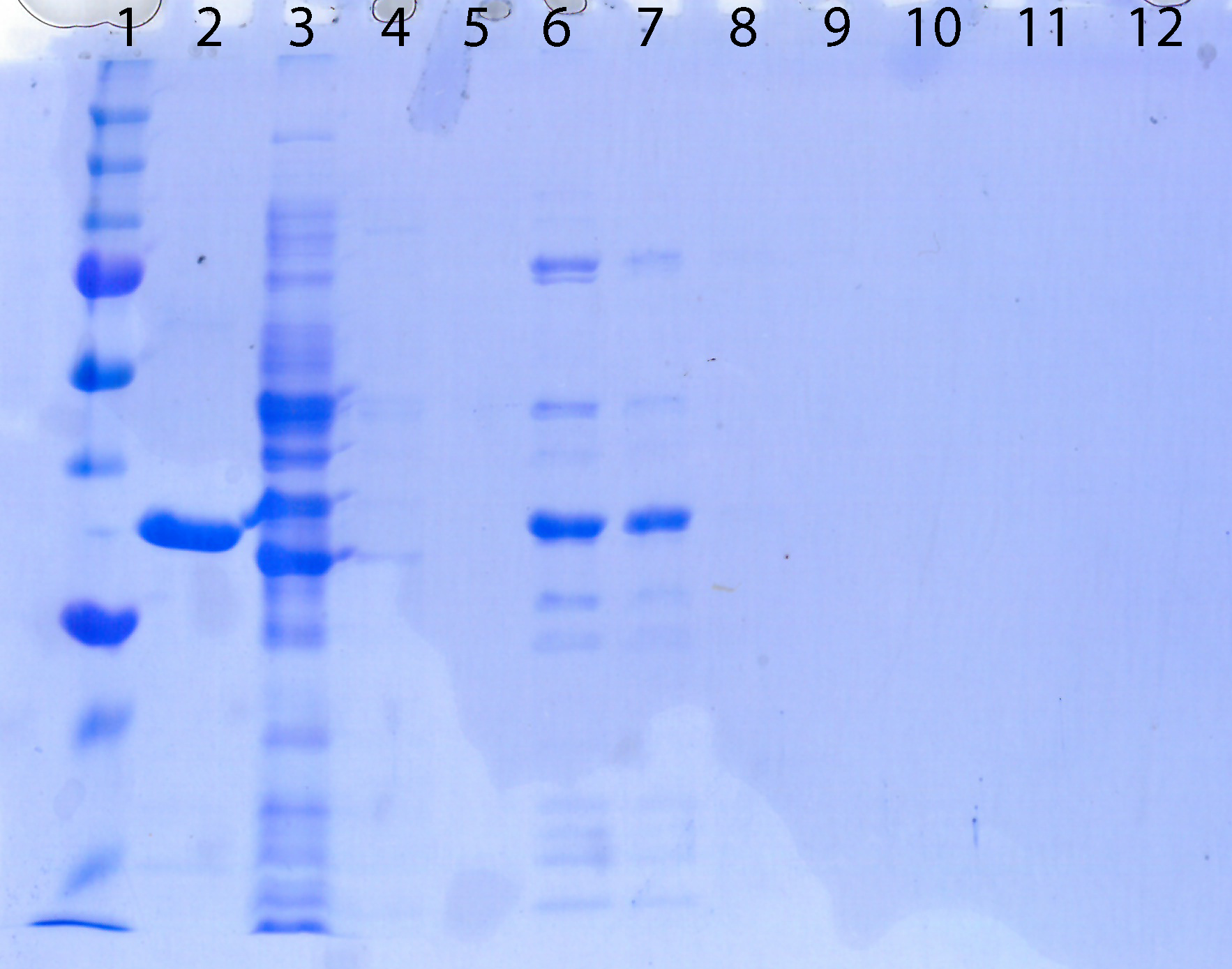


75 kDa -

50 kDa -

37 kDa -

25 kDa -

20 kDa -

**SDS-PAGE analysis of affinity purification of His_6_-CalB.**


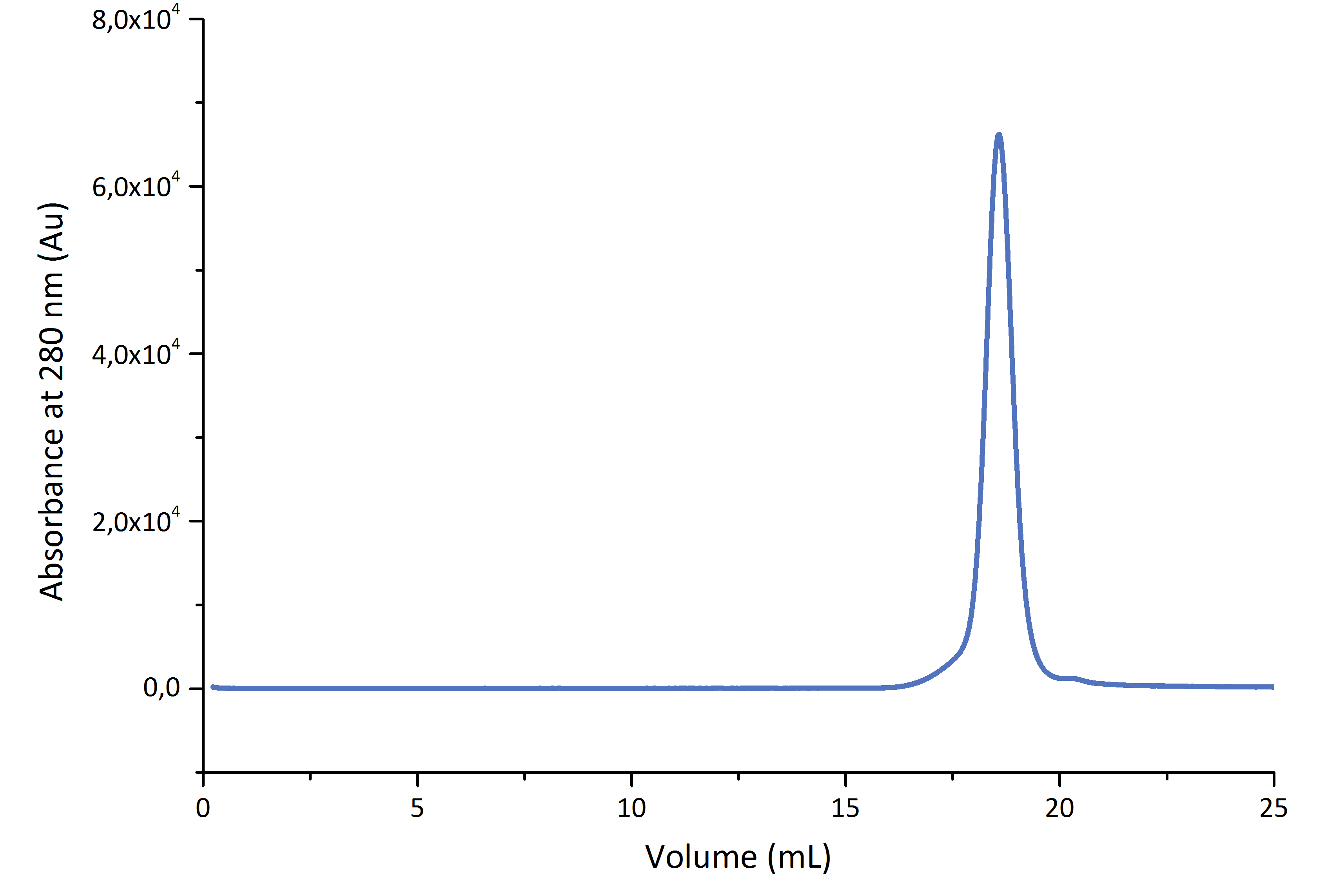


**Size exclusion chromatogram of purified His_6_-CalB in CalB buffer.**


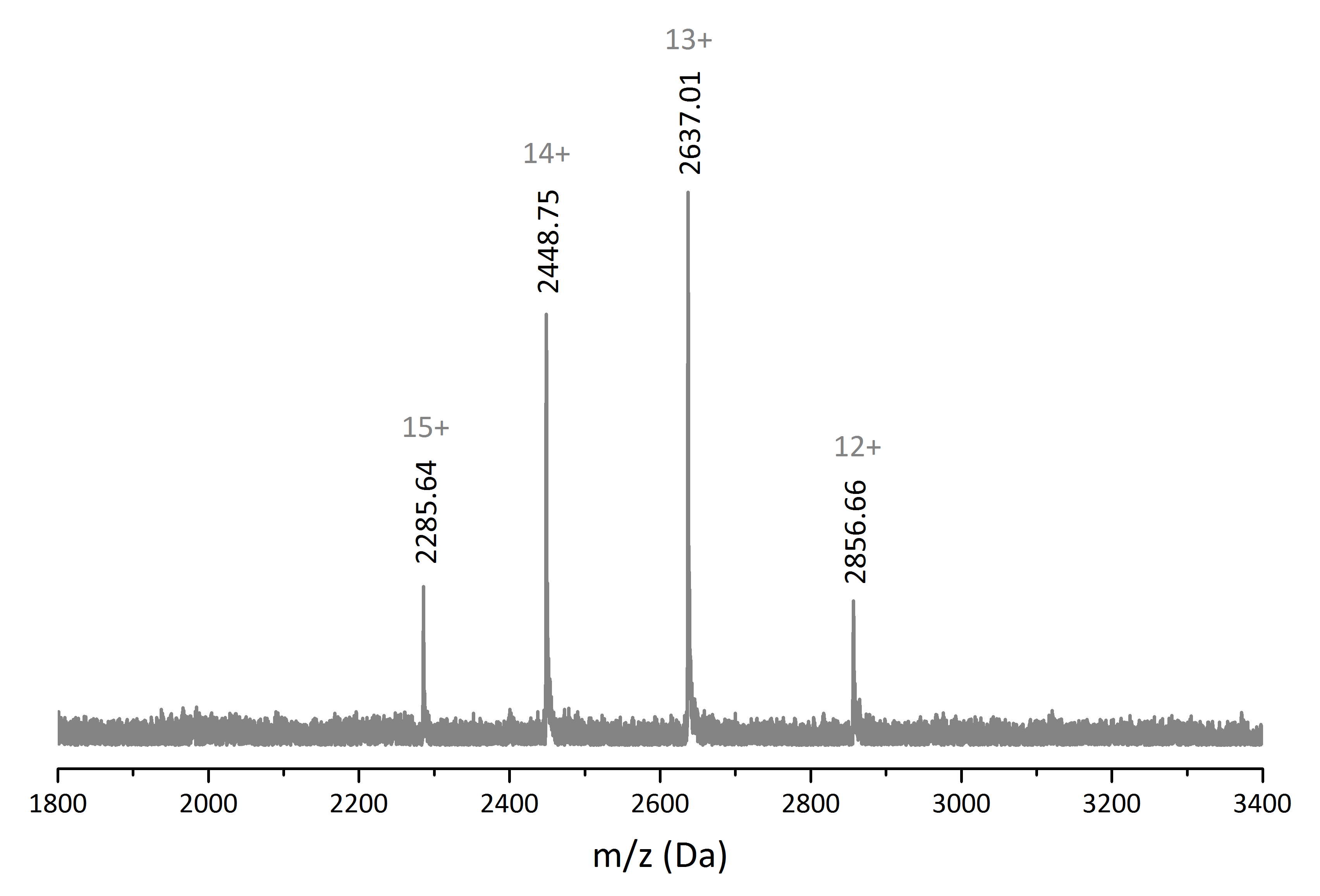

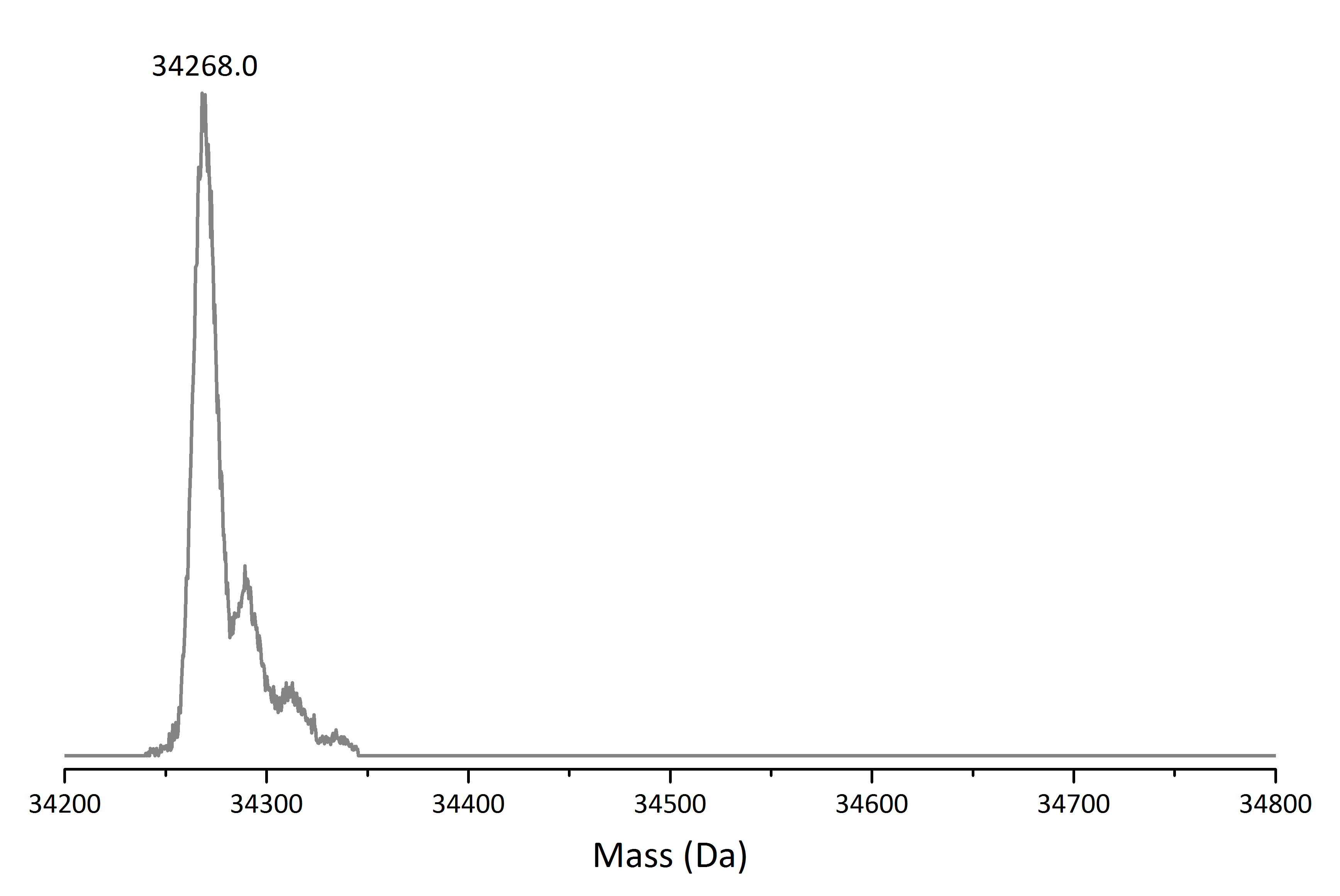


**ESI-TOF mass spectrometry of purified His_6_-CalB.** Deconvoluted total mass spectrum and multiply charged ion series (inset). The expected molecular weight is 34269.7 Da.

**1.6 Oligo and protein sequences**

**Table S1 - Amino acid** sequences of the proteins used in this study.

| **Name** | **Sequence** |
| --- | --- |
| His_6_-GFP | SVSKGEELFTGVVPILVELDGDVNGHKFSVSGEGEGDATYGKLTLKFICTTGKLPVPWPTLVTTLTYGVQCFSRYPDHMKQHDFFKSAMPEGYVQERTIFFKDDGNYKTRAEVKFEGDTLVNRIELKGIDFKEDGNILGHKLEYNYNSHNVYIMADKQKNGIKVNFKIRHNIEDGSVQLADHYQQNTPIGDGPVLLPDNHYLSTQSALSKDPNEKRDHMVLLEFVTAAGITLGMDELYKLPETGENLYFQSLEHHHHHH |
| His_6_-ELP-CCMV | GHHHHHHVPGVGVPGLGVPGVGVPGLGVPGVGVPGLGVPGGGVPGVGVPGLGLEVVQPVIVEPIASGQGKAIKAWTGYSVSKWTASCAAAEAKVTSAITISLPNELSSERNKQLKVGRVLLWLGLLPSVSGTVKSCVTETQTTAAASFQVALAVADNSKDVVAAMYPEAFKGITLEQLTADLTIYLYSSAALTEGDVIVHLEVEHVRPTFDDSFTPVY |
| wild type CCMV | MSTVGTGKLTRAQRRAAARKNKRNTRVVQPVIVEPIASGQGKAIKAWTGYSVSKWTASCAAAEAKVTSAITISLPNELSSERNKQLKVGRVLLWLGLLPSVSGTVKSCVTETQTTAAASFQVALAVADNSKDVVAAMYPEAFKGITLEQLTADLTIYLYSSAALTEGDVIVHLEVEHVRPTFDDSFTPVY |
| His_6_-PAMO | MGSSHHHHHHSSGLVPRGSHMLPKLVITHRVHEEILQLLAPHCELITNQTDSTLTREEILRRCRDAQAMMAFMPDRVDADFLQACPELRVIGCALKGFDNFDVDACTARGVWLTFVPDLLTVPTAELAIGLAVGLGRHLRAADAFVRSGKFRGWQPRFYGTGLDNATVGFLGMGAIGLAMADRLQGWGATLQYHARKALDTQTEQRLGLRQVACSELFASSDFILLALPLNADTLHLVNAELLALVRPGALLVNPCRGSVVDEAAVLAALERGQLGGYAADVFEMEDWARADRPQQIDPALLAHPNTLFTPHIGSAVRAVRLEIERCAAQNILQALAGERPINAVNRLPKANPAADSRSAAGMAGQTTVDSRRQPPEEVDVLVVGAGFSGLYALYRLRELGRSVHVIETAGDVGGVWYWNRYPGARCDIESIEYCYSFSEEVLQEWNWTERYASQPEILRYINFVADKFDLRSGITFHTTVTAAAFDEATNTWTVDTNHGDRIRARYLIMASGQLSVPQLPNFPGLKDFAGNLYHTGNWPHEPVDFSGQRVGVIGTGSSGIQVSPQIAKQAAELFVFQRTPHFAVPARNAPLDPEFLADLKKRYAEFREESRNTPGGTHRYQGPKSALEVSDEELVETLERYWQEGGPDILAAYRDILRDRDANERVAEFIRNKIRNTVRDPEVAERLVPKGYPFGTKRLILEIDYYEMFNRDNVHLVDTLSAPIETITPRGVRTSEREYELDSLVLATGFDALTGALFKIDIRGVGNVALKEKWAAGPRTYLGLSTAGFPNLFFIAGPGSPSALSNMLVSIEQHVEWVTDHIAYMFKNGLTRSEAVLEKEDEWVEHVNEIADETLYPMTASWYTGANVPGKPRVFMLYVGGFHRYRQICDEVAAKGYEGFVLT |
| His_6_-CalB | MGLPSGSDPAFSQPKSVLDAGLTCQGASPSSVSKPILLVPGTGTTGPQSFDSNWIPLSAQLGYTPCWISPPPFMLNDTQVNTEYMVNAITTLYAGSGNNKLPVLTWSQGGLVAQWGLTFFPSIRSKVDRLMAFAPDYKGTVLAGPLDALAVSAPSVWQQTTGSALTTALRNAGGLTQIVPTTNLYSATDEIVQPQVSNSPLDSSYLFNGKNVQAQAVCGPLFVIDHAGSLTSQFSYVVGRSALRSTTGQARSADYGITDCNPLPANDLTPEQKVAAAALLAPAAAAIVAGPKQNCEPDLMPYARPFAVGKRTCSGIVTPLEHHHHHH |

**1.7 Spectrophotometric activity assays**

**Experiments with His_6_-GFP**

Buffer: PBS buffer, PBS buffer with NaCl concentrations of 750-2000 mM

**Experiments with preceding NiCl_2_ incubation**

Experimental: NiCl_2_ (0 µM / 202 µM / 2.02 mM / 20.2 mM in buffer, 60 µL) was added to the catalyst (202.0 µM in buffer, 60 µL) in triplo. The mixtures were incubated at room temperature for 1 h. Then, the catalyst solution was added to the substrate as described in the general protocol.

Buffers: dimer buffer or salt-induced assembly buffer without EDTA, PBS buffer

**Experiments with different substrate types**

Experimental: To the reactions with the peroxidase substrates ABTS and *o*-PD, 1.0 % w/w H_2_O_2_ was added to the substrates prior to the addition of the catalyst. Extinction coefficients were taken from previous studies.^1-5^

Buffer: PBS buffer

Substrates: *p*-nitrophenyl acetate (*p*-NPA), *p*-nitrophenyl butyrate (*p*-NPB), MeO-PEG-NHCO-C_2_H_4_-CO-*p*-NP (*p*-NPA-PEG), carboxyfluorescein diacetate (CFDA), *p*-nitrophenyl phosphate (*p*-NPP), _L_-alanine 4-nitroanilide (Ala-pNA), *N*-succinyl- _L_-alanyl- _L_-alanyl- _L_-prolyl- _L_-phenylalanine 4-nitroanilide (Su-AAPF-pNA), 2,2′-azino-bis(3-ethylbenzothiazoline-6-sulfonic acid) (ABTS), *o*-phenylenediamine (*o*-PD)

Measured wavelengths: 410 nm (*p*-NPA, *p*-NPB, *p*-NPA-PEG, CFDA, *p*-NPP, Ala-pNA, Su-AAPF-pNA, ABTS), 490 nm (*o*-PD)

**Experiments with His_6_-ELP-CCMV and wt CCMV**

Buffers: dimer buffer, dimer buffer with NaCl concentrations of 750-1750 mM, salt-induced assembly buffer

**Experiments with His_6_-PAMO**

Buffer: PAMO buffer supplemented with NADPH (10 mM)

Substrates: *p*-NPA, *p*-nitroacetophenone

**Experiments with His_6_-CalB**

Experimental: a final catalyst concentration of 1.0 µM was used, as the reaction was too fast to determine the slope otherwise.

Buffer: PBS buffer

2 Supplemental figures


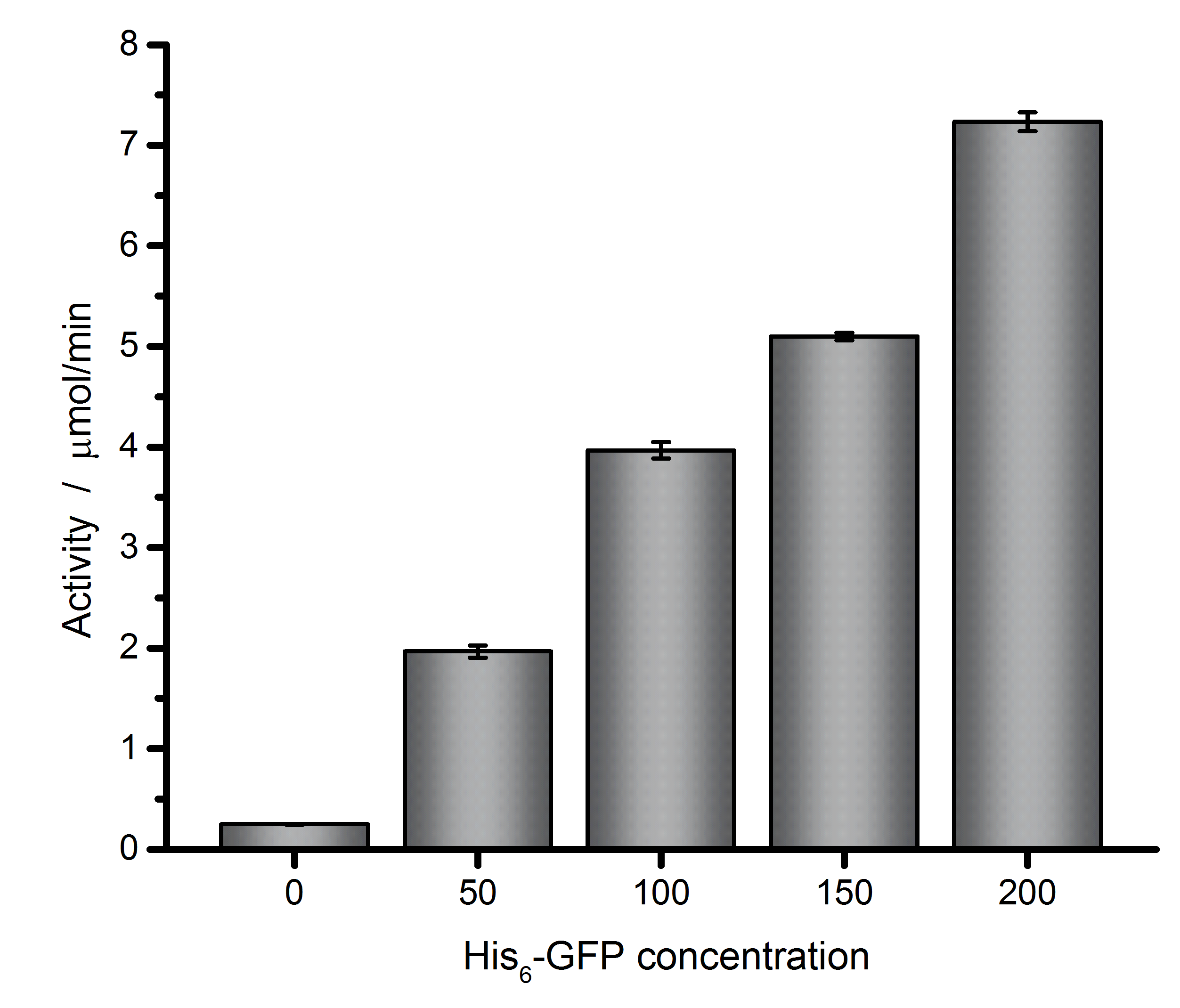

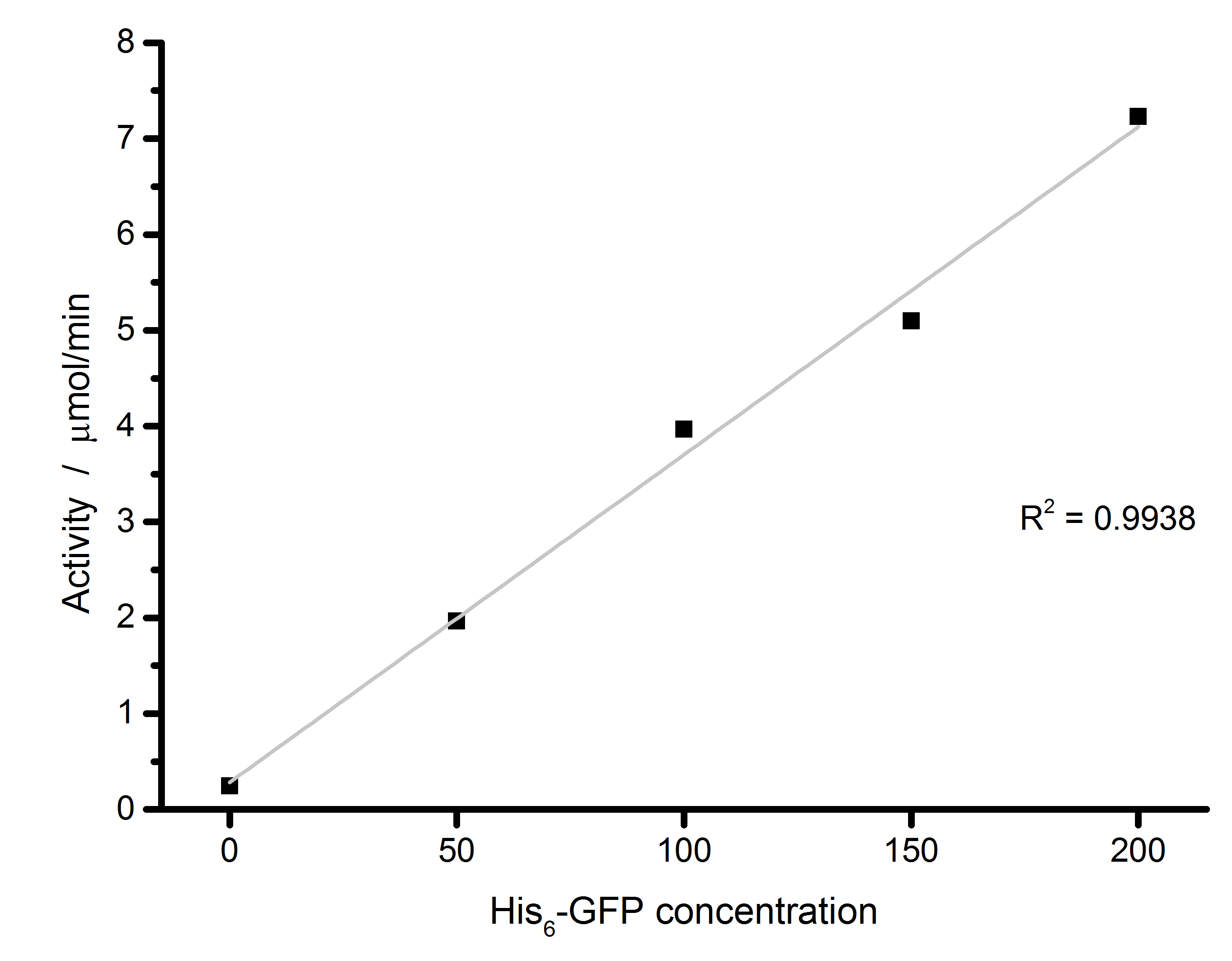


**Figure S1.** Activity of different concentrations of His_6_-GFP in the conversion of *p*-NPA to *p*-NP.


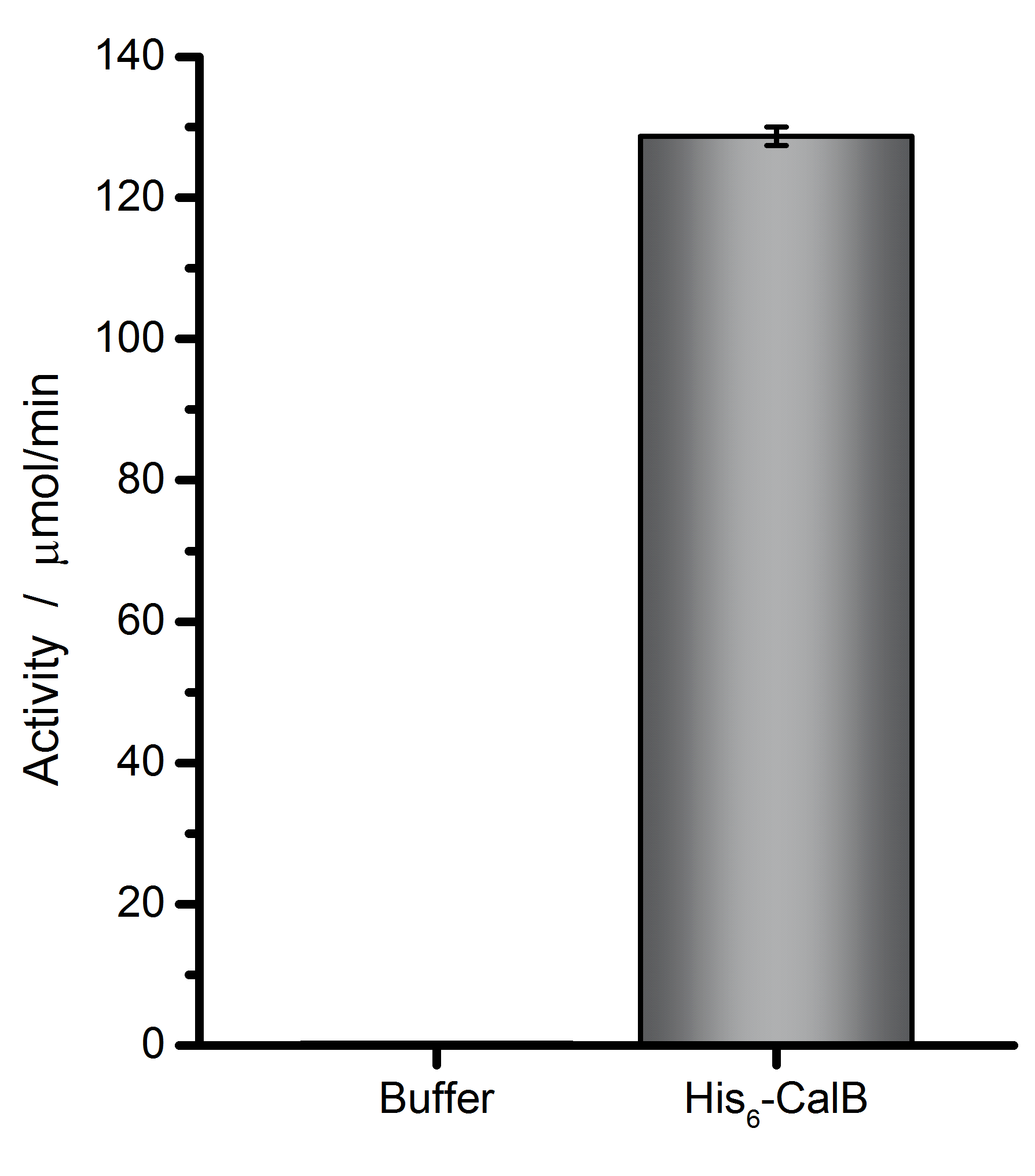


**Figure S2.** Activity of His_6_-CalB in the conversion of *p*-NPA to *p*-NP.


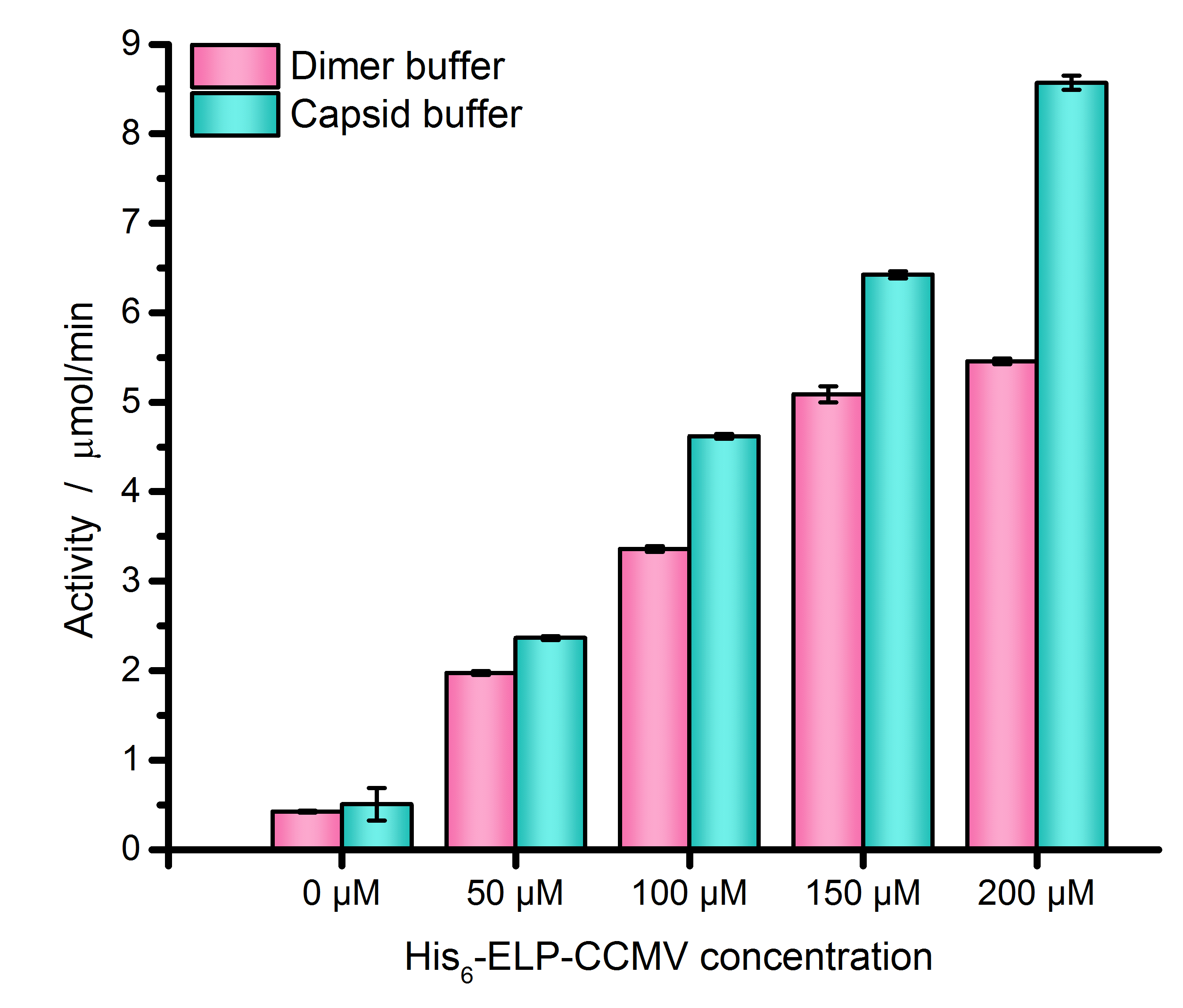

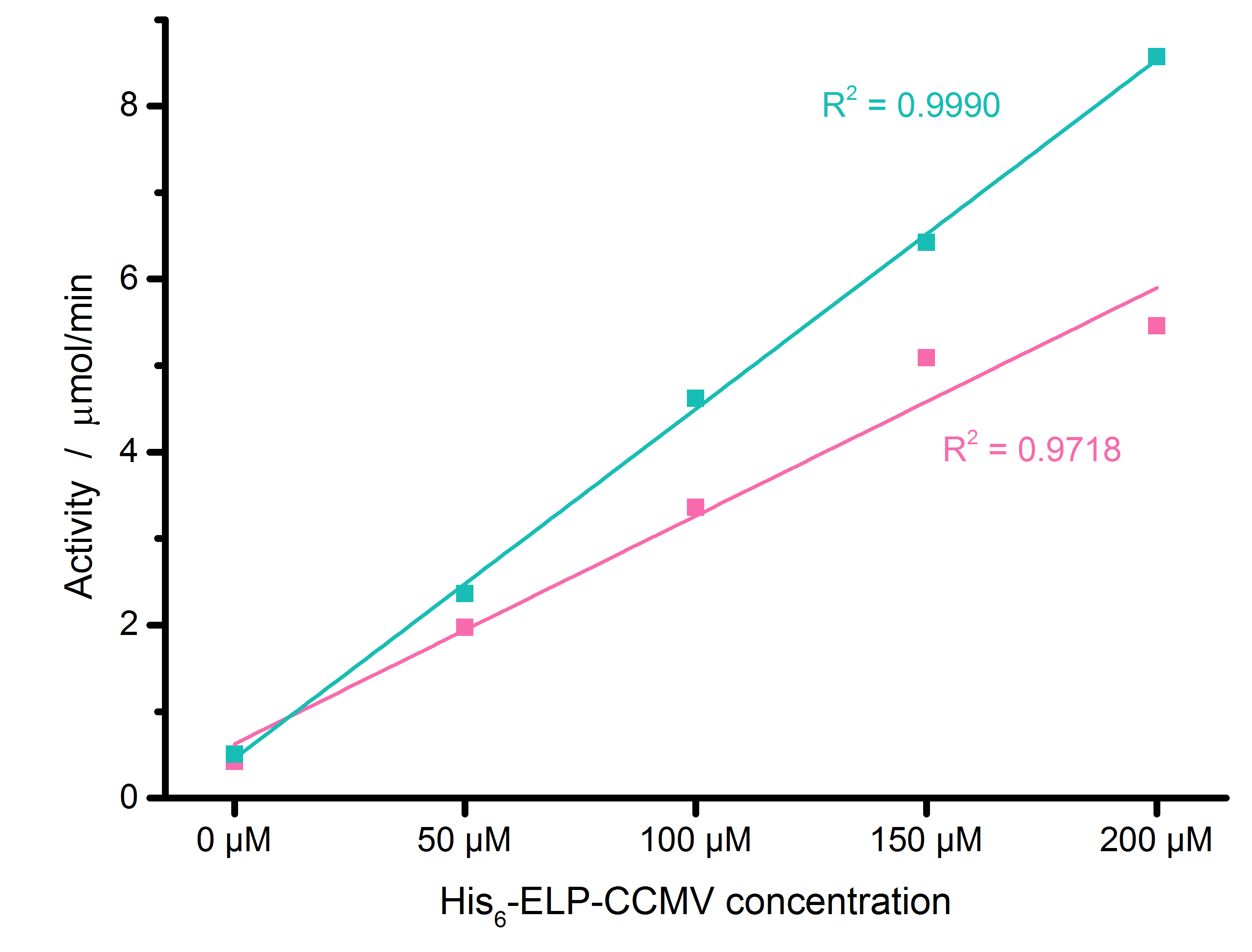


**Figure S3.** Activity of different concentrations of His_6_-ELP-CCMV in dimer buffer and capsid buffer in the conversion of *p*-NPA to *p*-NP.


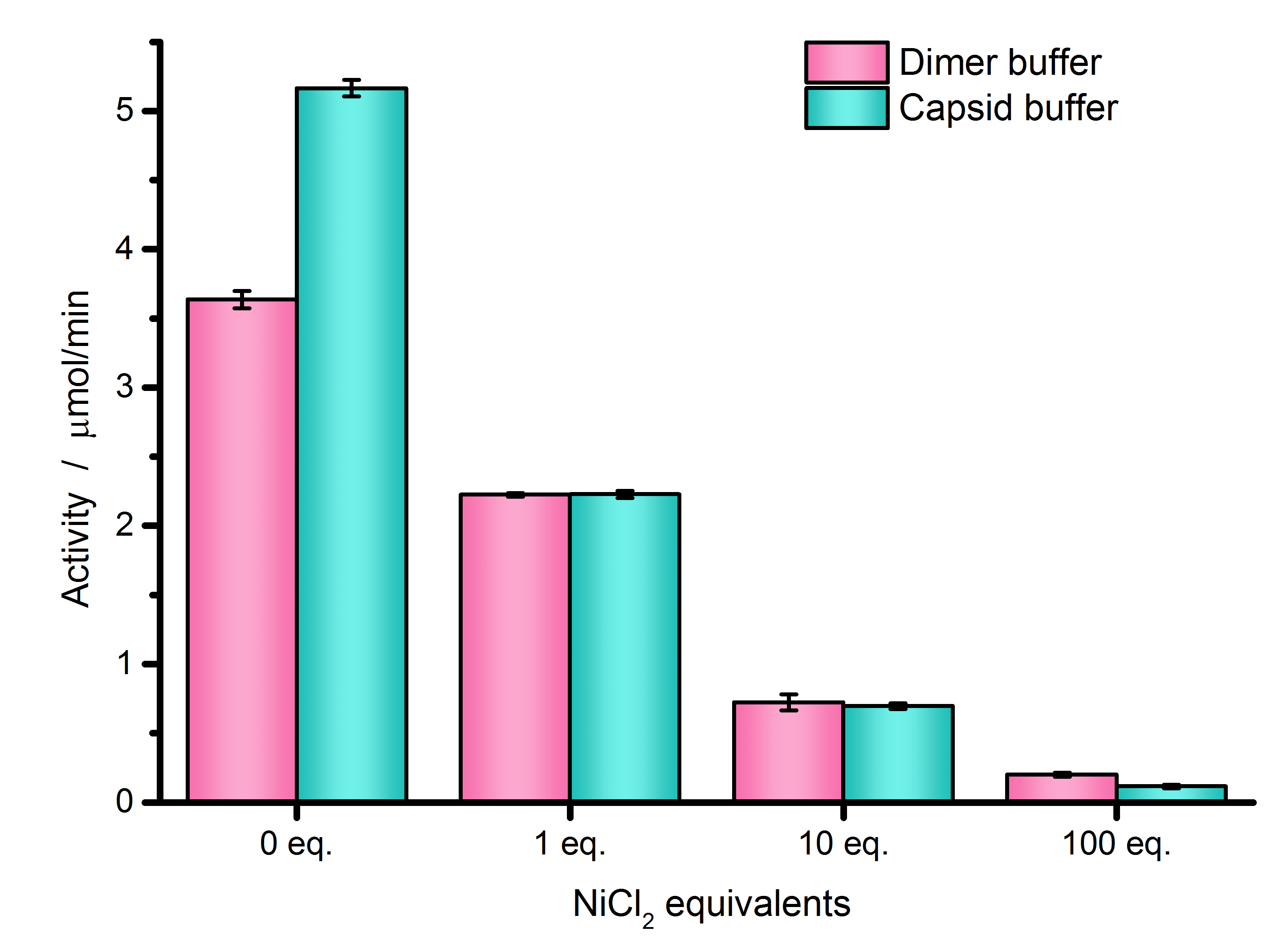


**Figure S4.** Activity of His_6_-ELP-CCMV in both dimer and capsid buffer after incubation with different equivalents of NiCl_2_. The conversion of *p*-NPA to *p*-NP was measured. Note: upon incubation of His_6_-ELP-CCMV with NiCl_2_ in dimer buffer, capsids will be formed.^6^


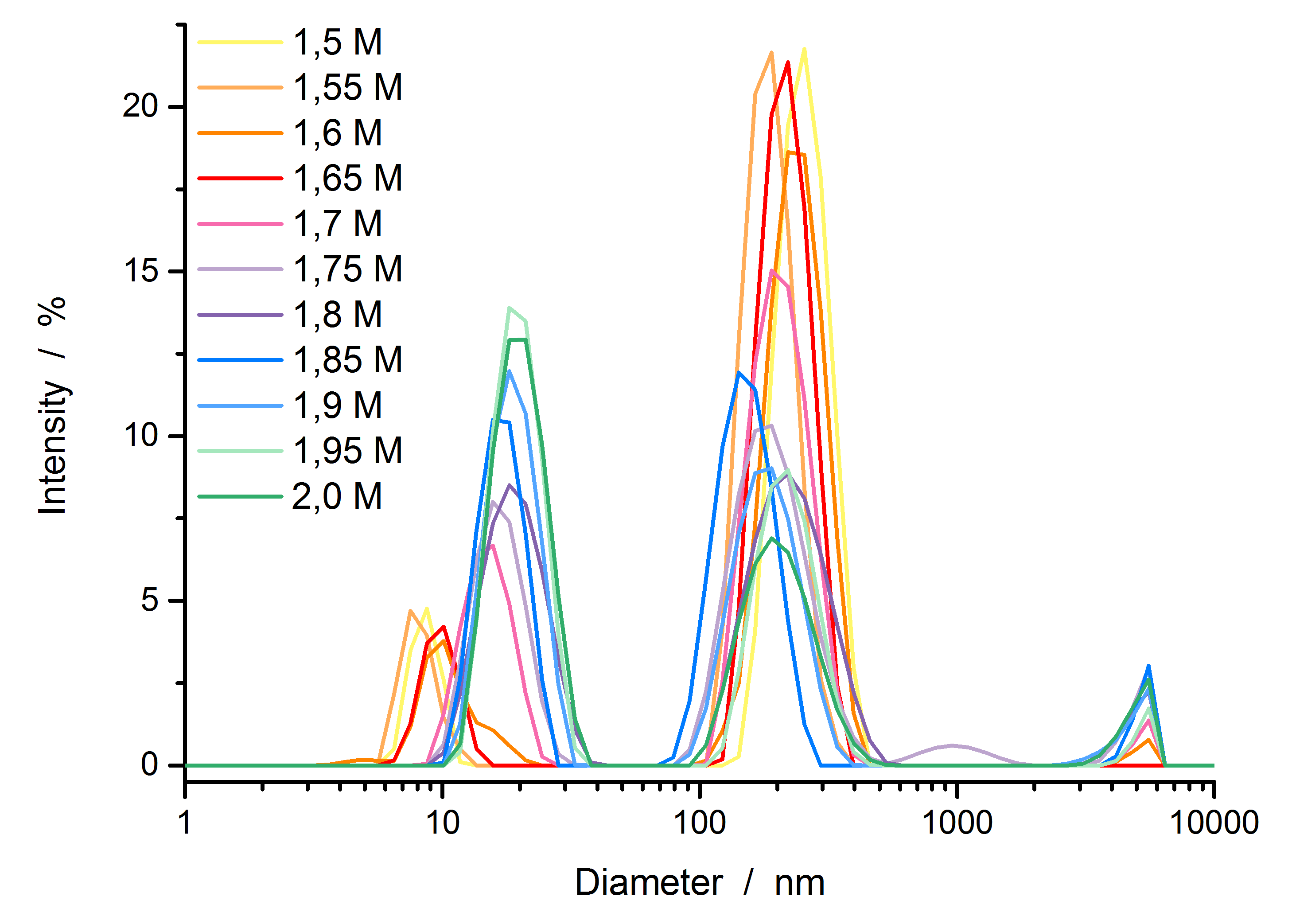


**Figure S5.** DLS analysis of His_6_-ELP-CCMV in dimer buffer without EDTA, with increasing NaCl concentrations. All graphs are an average of triplo measurements. Capsids are observed around 20 nm.


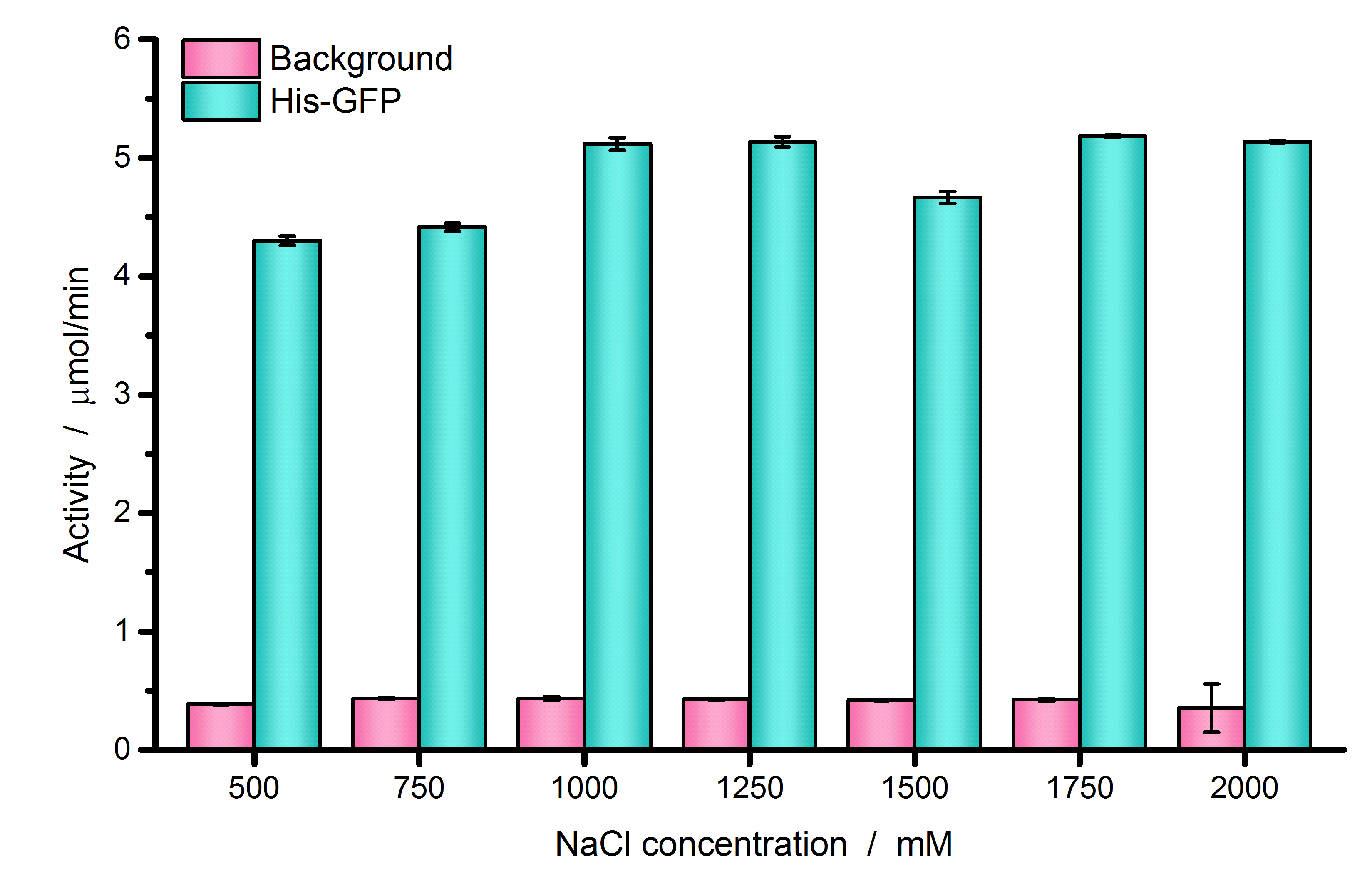


**Figure S6.** Activity of His_6_-GFP in dimer buffer with increasing NaCl concentrations. The conversion of *p*-NPA to *p*-NP was measured.


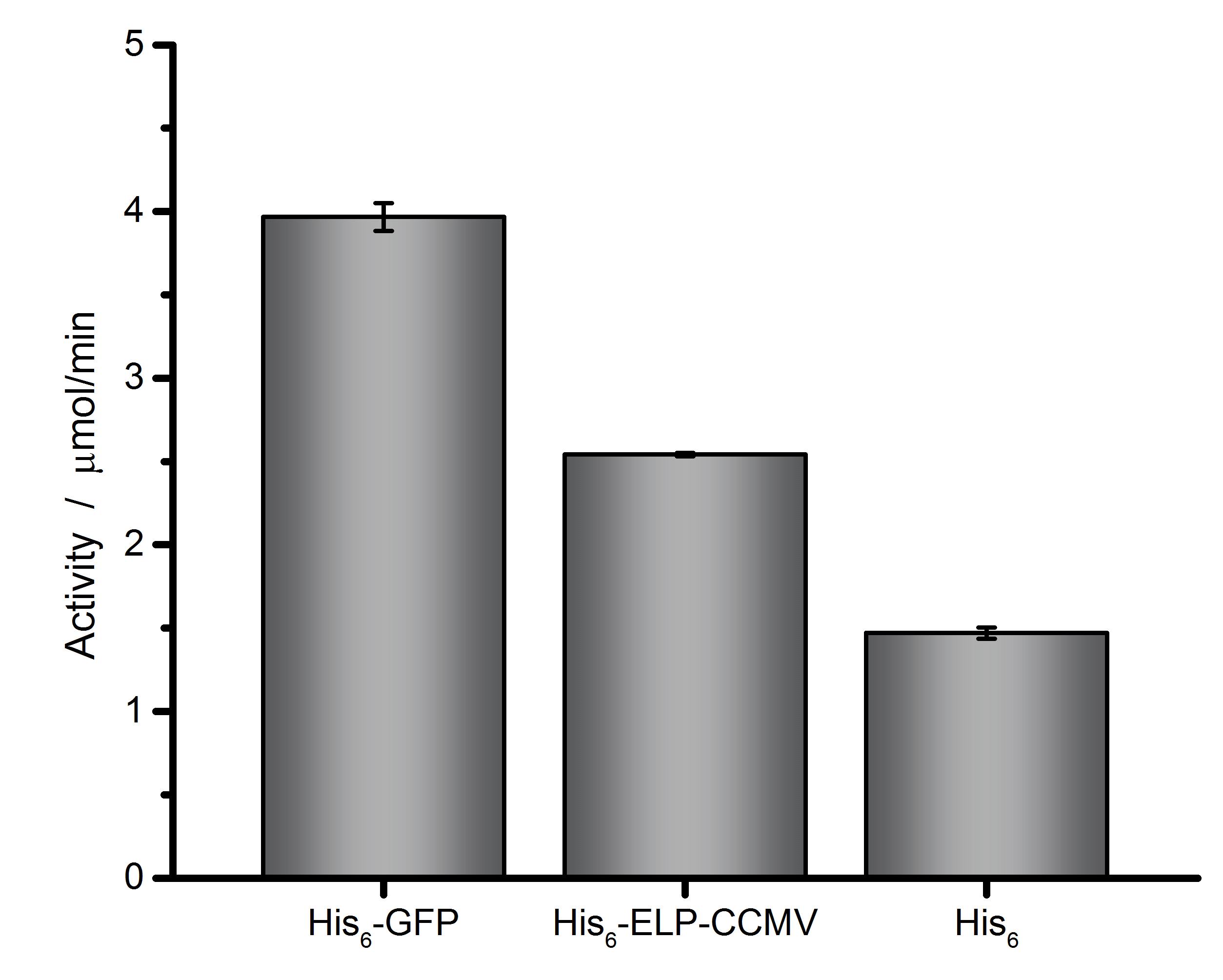


**Figure S7.** Activity of His_6_-GFP, His_6_-ELP-CCMV and His_6_ (data was combined from Figures 1, 4 and 5 from the manuscript). The conversion of *p*-NPA to *p*-NP was measured.

**Figure S8.** Activity of His_6_-PAMO in PBS buffer after incubation with 0 and 10 equivalents of NiCl_2_. The conversion of *p*-NPA to *p*-NP was measured.

3 References

(1) Heinzkill, M., Bech, L., Halkier, T., Schneider, P., and Anke, T. (1998) Characterization of laccases and peroxidases from wood-rotting fungi (family Coprinaceae). *Appl. Environ. Microbiol.* *64*, 1601–1606.

(2) Erlanger, B. F., Kokowsky, N., and Cohen, W. (1961) The preparation and properties of two new chromogenic substrates of trypsin. *Arch. Biochem. Biophys.* *95*, 271–278.

(3) Ignatenko, O. V, Gazaryan, I. G., Mareeva, E. A., Chubar, T. A., Fechina, V. A., Savitsky, P. A., Rojkova, A. M., and Tishkov, V. I. (2000) Catalytic properties of tryptophanless recombinant horseradish peroxidase. *Biochemistry (Moscow)* *65*, 583–587.

(4) Mota, M. C., Carvalho, P., Ramalho, J., and Leite, E. (1991) Spectrophotometric analysis of sodium fluorescein aqueous solutions. Determination of molar absorption coefficient. *Int. Ophthalmol.* *15*, 321–326.

(5) Couturier, M., Feliu, J., Haon, M., Navarro, D., Lesage-Meessen, L., Coutinho, P. M., and Berrin, J.-G. (2011) A thermostable GH45 endoglucanase from yeast: impact of its atypical multimodularity on activity. *Microb. Cell Fact.* *10*, 103.

(6) van Eldijk, M. B., Schoonen, L., Cornelissen, J. J. L. M., Nolte, R. J. M., and van Hest, J. C. M. (2016) Metal Ion-Induced Self-Assembly of a Multi-Responsive Block Copolypeptide into Well-Defined Nanocapsules. *Small* *12*, 2476–2483.
